# Inadequacy of existing clinical prediction models for predicting mortality after transcatheter aortic valve implantation

**DOI:** 10.1016/j.ahj.2016.10.020

**Published:** 2017-02

**Authors:** Glen P. Martin, Matthew Sperrin, Peter F. Ludman, Mark A. de Belder, Chris P. Gale, William D. Toff, Neil E. Moat, Uday Trivedi, Iain Buchan, Mamas A. Mamas

**Affiliations:** a Health e-Research Centre, University of Manchester, Manchester, United Kingdom; b Queen Elizabeth Hospital, Birmingham, United Kingdom; c James Cook University Hospital, Middlesbrough, United Kingdom; d MRC Bioinformatics Unit, Leeds Institute of Cardiovascular and Metabolic Medicine, University of Leeds; e Department of Cardiovascular Sciences, University of Leicester, Clinical Sciences Wing, Glenfield General Hospital, Leicester, United Kingdom; f NIHR Leicester Cardiovascular Biomedical Research Unit, Leicester, United Kingdom; g Royal Brompton and Harefield National Health Service (NHS) Foundation Trust, London, United Kingdom; h Sussex Cardiac Centre, Brighton and Sussex University Hospitals, Brighton,United Kingdom; i Keele Cardiovascular Research Group, Keele University, Stoke-on-Trent, UK, Royal Stoke Hospital, University Hospitals North Midlands, Stoke-on-Trent, United Kingdom

## Abstract

**Background:**

The performance of emerging transcatheter aortic valve implantation (TAVI) clinical prediction models (CPMs) in national TAVI cohorts distinct from those where they have been derived is unknown. This study aimed to investigate the performance of the German Aortic Valve, FRANCE-2, OBSERVANT and American College of Cardiology (ACC) TAVI CPMs compared with the performance of historic cardiac CPMs such as the EuroSCORE and STS-PROM, in a large national TAVI registry.

**Methods:**

The calibration and discrimination of each CPM were analyzed in 6676 patients from the UK TAVI registry, as a whole cohort and across several subgroups. Strata included gender, diabetes status, access route, and valve type. Furthermore, the amount of agreement in risk classification between each of the considered CPMs was analyzed at an individual patient level.

**Results:**

The observed 30-day mortality rate was 5.4%. In the whole cohort, the majority of CPMs over-estimated the risk of 30-day mortality, although the mean ACC score (5.2%) approximately matched the observed mortality rate. The areas under ROC curve were between 0.57 for OBSERVANT and 0.64 for ACC. Risk classification agreement was low across all models, with Fleiss's kappa values between 0.17 and 0.50.

**Conclusions:**

Although the FRANCE-2 and ACC models outperformed all other CPMs, the performance of current TAVI-CPMs was low when applied to an independent cohort of TAVI patients. Hence, TAVI specific CPMs need to be derived outside populations previously used for model derivation, either by adapting existing CPMs or developing new risk scores in large national registries.

Despite surgical aortic valve replacement (SAVR) being the definitive treatment strategy for severe symptomatic aortic stenosis, a significant proportion of patients are not offered surgery due to co-morbidities or frailty that contribute to high surgical risks and adverse outcomes in such patient groups.^1^ Transcatheter aortic valve implantation (TAVI) has emerged as an efficacious but less invasive treatment option in high and intermediate operative risk patients.2., 3., 4., 5. As such, treatment allocation between medical management, SAVR and TAVI depends on multiple factors, but key is the assessment of the patient's procedural risk. Clinical prediction models (CPMs), which quantify the risks associated with the proposed treatment strategy at an individual patient level, can aid heart-teams in this clinical decision-making process and are vital for audit purposes between TAVI centers.

Cardiac surgery CPMs for short-term mortality prediction, such as the EuroSCORE6., 7. and the Society of Thoracic Surgeons Predicted Risk of Mortality (STS) model,^8^ have been used to identify high-risk patients in randomized trials of TAVI.2., 3. However, these surgical CPMs perform poorly in predicting risk after both SAVR and TAVI, as exemplified in the PARTNER cohort A trial where there was large disagreement between the observed and STS-expected 30-day mortality.^3^ Moreover, several cohort studies have shown the inaccuracy of the surgical CPMs in predicting mortality after TAVI.9., 10., 11.

Consequently, TAVI specific CPMs are beginning to emerge from large cohorts of TAVI patients.12., 13., 14., 15. In particular, the German Aortic Valve Score (German AV) was developed using patients who underwent either surgical replacement or TAVI,^13^ while TAVI-specific CPMs have been derived in the France TAVI registry (FRANCE-2 model),^14^ the Italian TAVI registry (OBSERVANT model)^12^ and the Society of Thoracic Surgeons/American College of Cardiology Transcatheter Valve Therapy registry (ACC model).^15^ However, the performance of the aforementioned TAVI-CPMs in large cohorts of patients outside of their derivation cohorts is unknown. Hence, it is unclear if they can be reliably used in other national settings.

Therefore, the aim of this study was to investigate the performance and agreement of the German AV, FRANCE-2, OBSERVANT and ACC TAVI-CPMs for predicting 30-day mortality outside their development cohorts, to examine if the performance was sufficient for them to be used for this purpose. The study compared the TAVI-CPM performance against surgical CPMs, namely the Logistic EuroSCORE (LES), EuroSCORE II (ESII) and STS score.

## Methods

### UK TAVI registry

Prospectively collected data on every TAVI procedure in the United Kingdom from January 2007 to December 2014 were obtained through the UK TAVI registry.^16^ By the end of 2014, 34 UK centers were performing TAVI procedures with multi-disciplinary teams of cardiologists, surgeons and other healthcare professionals at each center deciding on patients' suitability for TAVI.^16^ The Web-based registry comprises 95 variables detailing patient demographics, risk factors for intervention, procedural details and adverse outcomes up to the time of hospital discharge. All-cause mortality tracking was obtained from the Office for National Statistics providing the life-status of English and Welsh patients (two countries of the UK). Mortality tracking was unavailable for patients in Northern Ireland and Scotland and, as such, these patients were removed from the analysis.

### Statistical analysis

Multiple imputation was used for missing values, with ten datasets imputed.^17^ Missing life-status was not imputed and this analysis excluded any patient who had such a missing endpoint. To avoid underestimation of covariate-outcome associations, 30-day mortality indication was used in the imputation models for missing covariates.^18^ Further details of the imputation procedure are given in the supplementary material.

The risk of 30-day mortality implied by each CPM was retrospectively calculated for each patient based on the published regression coefficients.6., 7., 8., 12., 13., 14., 15. This analysis used clinical reasoning to make assumptions regarding translation between variable definitions in the published CPMs and those in the UK TAVI dataset. Any CPM risk-prediction variable that was not recorded in the UK TAVI registry was assumed risk factor absent for all patients. The full translation between each CPM and the TAVI registry variables is given in the supplementary material (Supplementary Tables I–VII) along with the statistical code used to calculate the scores.

The performance of each CPM was assessed in terms of calibration and discrimination. Calibration is the agreement between the expected and observed event rates across the full risk range; discrimination is the ability of the CPM to distinguish between those who will experience an event and those who will not. Discrimination of the risk models was analyzed using the area under the receiver operating characteristic (ROC) curve, with values between 0.5 and 1 where higher values indicate better discrimination. To examine the calibration of each CPM, a logistic regression model was fitted with the event indicator as the outcome and the linear predictor from the CPM as the only covariate.^19^ Perfect calibration would occur when the corresponding intercept and slope are zero and one respectively, with the intercept estimated assuming a slope of unity. Furthermore, the Brier Score was used as a measure of overall performance, with values between 0 (perfect prediction) and 1 (worst prediction).^20^ CPM performance was analyzed in the whole cohort and within several subgroups. The following subgroups were considered: age (≤ or >75), sex, diabetes status, access route (transfemoral vs non-transfemoral), valve type (SAPIEN vs CoreValve), previous coronary artery bypass graft status, left ventricular (LV) function (LV ejection fraction [LVEF] <50% or LVEF ≥50%), and procedure urgency (elective vs non-elective).

Patient-level risk agreement between CPMs was analyzed in the surgical models and the TAVI models separately to facilitate fair comparisons. It was decided, a priori, to derive cut-off values for each CPM that defined three risk levels (low-, medium- and high-risk), with approximately equal patient numbers in each. The proportions of patients for whom risk classification agreed between multiple CPMs was then calculated. In addition, Fleiss's κ was calculated in the surgical and TAVI models.^21^ A sensitivity analysis was conducted in which the risk stratifications were re-defined to give a population ratio of 1:3:1 for low, medium, and high risk, respectively.

R version 3.3.1^22^ was used for all statistical analyses. Multiple imputation of the dataset was completed using the mice package,^23^ graphical plots were made using the ggplot2 package^24^ and the package pROC was used for constructing ROC curves.^25^

The Health e-Research Centre, funded by the Medical Research Council [MR/K006665/1] and the North Staffordshire Heart Committee supported this work. The authors are solely responsible for the design and conduct of this study, all study analyses, the drafting and editing of the manuscript, and its final contents.

## Results

The UK TAVI registry included all 7431 patients who underwent a TAVI procedure between January 2007 and December 2014. All patients from Northern Ireland (n = 400) and the majority of Scottish patients (n = 193) were excluded from the analysis due to absence of Office for National Statistics mortality tracking. Out of the remaining 6838 patients, a further 162 were removed due to missing life status, leaving 6676 patients studied in this analysis. The observed survival rates were 94.6%, 83.3% and 64.4% at 30-day, 1-year, and 3-year follow-up, respectively. Table I presents summary statistics for baseline characteristics of the patients in the UK TAVI registry.

### Performance analysis

**Figure 1:**
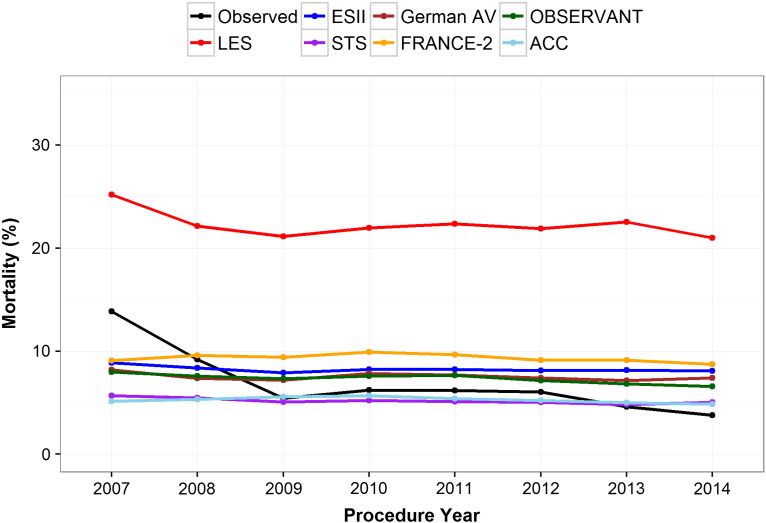
Temporal changes in observed and expected mortality over each of the CPMs.

From January 2007 to December 2014, there were 360 deaths within 30-days of the TAVI procedure (5.4%). The expected 30-day mortalities in the whole cohort were 21.9%, 8.1%, 5.1%, 7.4%, 9.2%, 7.1%, and 5.2% from the LES, ESII, STS, German AV, FRANCE-2, OBSERVANT, and ACC CPMs, respectively (Table II). The ACC score and STS model were the closest to the observed mortality in terms of absolute and relative differences, while the LES overestimated risk by a factor of four (Table II). After a decrease from 2007 to 2008, the observed 30-day mortality per year remained approximately constant, with further decreases in 2013 and 2014 (Figure 1). In contrast, the profile of the majority of CPMs remained approximately constant throughout (Figure 1). The inflated observed 30-day mortality in the first two years likely reflects the UK learning curve and advances in TAVI technology, while the CPMs do not account for such factors. The observed and expected 30-day mortality rates over each subgroup are given in Supplementary Table VIII.

Table III shows the performance of each CPM in the whole cohort. While the calibration intercepts of the ACC and STS models were significantly close to zero (ie, the observed and expected mortalities agreed), the 95% CIs for the calibration slopes did not span one, indicating model miscalibration. Poor discrimination was observed, with area under the ROC curves between 0.57 and 0.64 for the whole cohort; the FRANCE-2 TAVI score and the ACC TAVI score had the highest AUC values of 0.62 and 0.64, respectively. Overall performance, as measure by the Brier score, was similar for the majority of models with values of 0.05; a Brier score of 0.09 for the LES was the highest (worst) amongst the models. Quantitatively similar results were obtained from a sensitivity analysis that excluded patients who underwent TAVI in 2007 or 2008 (n = 337) where the observed mortality was elevated over that in subsequent years (Supplementary Table IX).

The performances of all the CPMs in each subgroup are given in the supplementary material (Supplementary Table X). The expected mortality from the ACC TAVI model was significantly close to the observed mortality across all strata, but satisfactory calibration (calibration intercept and slope close to zero and one, respectively) was only observed for this CPM in female and diabetic subgroups. All other models were miscalibrated across strata. The area under the ROC curve was below 0.7 for all CPMs across the subgroups, with the majority close to 0.6; the ACC and FRANCE-2 CPMs had the highest discrimination across subgroups.

### Agreement analysis

**Figure 2:**
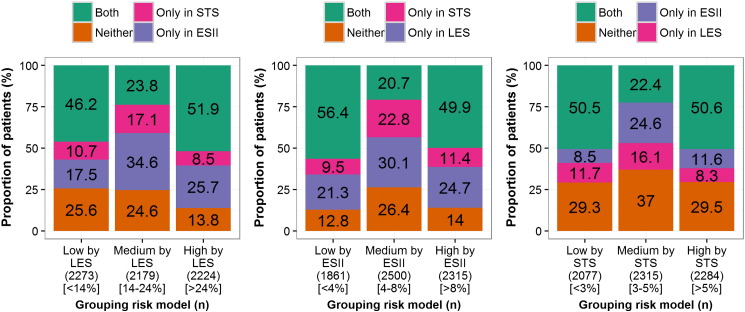
The proportion of patients that agree in risk allocation over the surgical based CPMs. Each bar represents a risk stratification by one of the surgical CPMs, with the segments of that bar showing the proportion of patients that were also grouped in that risk strata by none, one or both of the other surgical CPMs.

**Figure 3:**
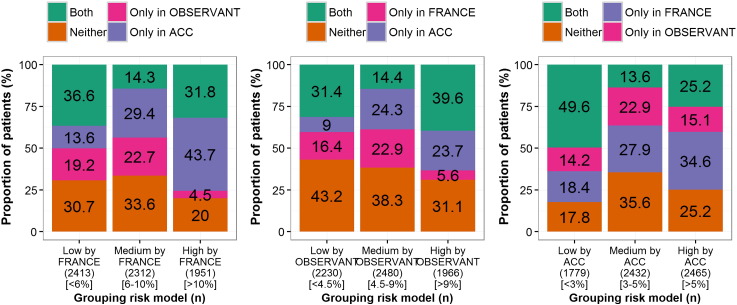
The proportion of patients that agree in risk allocation over the TAVI based CPMs. Each bar represents a risk stratification by one of the TAVI-CPMs, with the segments of that bar showing the proportion of patients that were also grouped in that risk strata by none, one or both of the other TAVI-CPMs.

The chosen cut-off values that gave approximately equal numbers of patients in low-, medium-, and high-risk categories are given in Table IV. Based on these cut-off values, the proportions of patients classified in each risk level who were similarly classified across the other CPMs were calculated (Figure 2 for the surgical based CPMs and Figure 3 for the TAVI based CPMs). A low level of agreement at an individual patient level was observed; for example, only 31.8% of the 1951 patients grouped as high-risk by FRANCE-2 >10% were also grouped as high-risk by the OBSERVANT and ACC models (Figure 3). Quantifying agreement between the CPMs using Fleiss's *κ*, highlighted that agreement between all the surgical scores was moderate (*κ* = 0.37), while that between all the TAVI models was poor (*κ* = 0.20). The pairwise Fleiss's *κ* values are given in Table IV, which shows that there was moderate agreement between the FRANCE-2 and ACC TAVI models (*κ* = 0.33). Risk stratifications were re-defined to give a population ratio of approximately 1:3:1 for low, medium, and high risk. Here, the results indicated marginally improved levels of agreement, but these were still moderate. Specifically, the Fleiss's κ across the surgical scores was 0.40 and that between the TAVI models was 0.20, with pairwise Fleiss's *κ* values given in Supplementary Table XI.

## Discussion

Clinical prediction models form the cornerstone of risk stratification for patients undergoing invasive procedures, helping to guide both treatment allocation and the consent process. However, their performance needs to be tested in large datasets independent to those in which the models were developed before they can be used in external populations.26., 27. Our analysis of the UK TAVI registry has systematically demonstrated that outside their development cohorts, the German AV, FRANCE-2, OBSERVANT and ACC TAVI CPMs are miscalibrated and have low discrimination at predicting 30-day mortality. These results support previous work in this area.^28^ In the current study, the FRANCE-2 and ACC models had the highest discrimination out of all those considered, with these comparing favorably to the internal validation results reported when these models were derived.14., 15. In addition, although the ACC model was miscalibrated, the expected mortality was significantly close to the observed mortality across all subgroups considered in this analysis. However, of note is that the ACC model was predominately developed to predict in-hospital mortality, which potentially contributes to the agreement between the observed and expected event rates for this model.

The performance of any CPM is expected to drop when they are applied in populations external to the development set since patient mix and procedure techniques vary between populations.26., 27., 29. Consequently, the findings of the current study are, perhaps, unsurprising given that the TAVI-CPMs achieved only moderate performance in their respective development datasets.12., 14., 15. Current TAVI cohorts predominantly represent a particularly high-risk and homogenous group of patients, potentially contributing to the lack of a highly predictive TAVI-CPM. Future TAVI-CPMs need to be developed by utilizing the contemporary large registries that are emerging, which will inevitably require greater harmonization between variable and outcome definitions amongst national datasets.

Moreover, many of the co-morbidities used in the development of CPMs are cardiovascular risk factors, with important non-cardiovascular co-morbidities not considered.^30^ In particular, frailty is not reflected in many of the CPMs, despite being particularly prevalent in elderly patients with aortic stenosis and previous work suggesting frailty to be associated with poor TAVI outcomes.31., 32. A CPM that aims to predict long-term mortality following TAVI found that the inclusion of frailty in their model significantly increased the discrimination.^33^ Similarly, a previously published CPM that aims to predict mortality and/or a decline in quality of life following TAVI included an indication of 6-minute walk test distance.^34^

The present study indicated that the 30-day mortality was elevated in 2007 and 2008 over that in subsequent years, but the sensitivity analysis that excluded 2007/08 procedures indicated similar results to the main analysis. Previous studies have shown a learning curve associated with TAVI, but center/operator volume and outcome relationships remain debated.35., 36., 37. Nevertheless, measures of operator volume or experience are not used in CPMs since accounting for such variables would be inappropriate, particularly when the purpose of a CPM might be to benchmark an individual operators/centers performance. Similarly, the addition of operator volume/experience in a CPM would make it almost impossible for a physician to convey the predicted risk to a patient.

### Comparison with performance of the surgical CPMs

The current study confirms previous work in showing that the performance of the LES, ESII and STS models are poor at predicting 30-day mortality post TAVI.9., 10., 11. The STS model outperformed the other surgical models, with the STS expected 30-day mortality rate not significantly different from the observed 30-day mortality rate. This finding has been previously observed9., 11. and is perhaps attributable to the fact the STS score has a specific model for isolated valve surgery.^8^ Of note, previous TAVI registries have reported mean STS values higher than that found in this study, perhaps due to the assumptions made in our study regarding the calculation of the STS model. For example, the FRANCE TAVI registry reported STS values of around 18%, while the Italian CoreValve registry reported values of 11%.38., 39.

Nonetheless, comparing the surgical CPMs to the TAVI-CPMs highlights that the latter performed better than the former when internally validated12., 14. and the current study shows that the FRANCE-2 and ACC models outperformed the surgical scores. Surgical CPMs are limited in their use in transcatheter procedures because they were derived from surgical populations. Not only are the procedural risks of TAVI different from those in SAVR, but there is lack of grading between the severities of co-morbidities in the surgical CPMs. For example, chronic obstructive pulmonary disease is a risk factor in LES, but there is no further distinction between the severity of chronic obstructive pulmonary disease or even other severe lung disease. Since the heart-team considers such severities when deciding between SAVR and TAVI, grading of co-morbidities should be included in future TAVI-CPMs.

### Patient-level agreement analysis

This study highlighted that the classification of patient risk varies between multiple CPMs, even when comparing surgical and TAVI based CPMs separately. A Pearson correlation coefficient of 0.56 has previously been reported between the LES and STS score,^10^ with similar correlation between these scores reported in other studies.^11^ Such an analysis does not necessarily indicate the level of agreement between two risk models, since the correlation is only assessing the linear relationship between them.^40^ Although the current study found higher agreement between the surgical models than between the TAVI models, this was driven by the ESII being an updated version of the LES. The lack of agreement between the scores further highlights previously published recommendations that risk assessment should be based on heart-team discussion in combination with multiple CPMs.^4^

### Limitations

A limitation of the current work is that assumptions were required when linking the definitions of model variables with the TAVI dataset, as described in the supplementary material. For example, the lowest LV function category in the ESII model is LVEF <20% whereas that in the UK TAVI dataset is LVEF<30%, with this analysis assuming these definitions to be equivalent. Such assumptions are an artifact of different recording practices between national registries. Accordingly, some of the surgical CPMs could not be calculated exactly as they were published, which could induce bias into the calculated predicted risks. This study used surrogate variables to mitigate this wherever possible and all assumptions were made to reflect the TAVI procedure as accurately as possible. As noted above, the calculated STS score in this study is lower than previously reported from other TAVI registries. Lack of variables including mitral valve, hypertension and severity of pulmonary disease could have contributed to this, but our findings compare favorably to previous work. Similarly, the assumption of risk factor absent for variables that were included in CPMs but not recorded in the UK TAVI registry (eg, mitral valve replacement or infective endocarditis) may induce bias, but any such bias is likely to be negligible given the variables where this assumption was needed.

### Implications for future work

Based on this work, the development of further TAVI-CPMs is recommended in populations of interest. Although there is an indication of the feasibility of TAVI in intermediate risk patients,^5^ TAVI-CPMs are still required, especially for procedure audit purposes and risk stratification analyses. Rather than developing new scores from scratch, model updating techniques could be applied to the current TAVI-CPMs to adapt them to new national cohorts.^41^ For instance, re-fitting the current models to the population of interest and/or the addition of new risk factors, such as frailty, could improve prediction.31., 42. Further work in this area is recommended. Secondly, developing TAVI models that predict both short- and long-term outcomes would be particularly valuable, especially if they included a measure of futility.

## Conclusions

The FRANCE-2 and ACC TAVI models had the highest performance across all CPMs considered. However, all the CPMs had low calibration and discrimination, reducing their suitability for risk stratification outside their development cohorts. Future iterations of existing TAVI models may benefit from including non-cardiovascular co-morbidities such as frailty. The derivation of TAVI-CPMs in contemporary large registries is recommended, but it remains to be determined if this is best achieved by updating/revising existing TAVI scores, by developing new CPMs in specific cohorts, or a combination of the two.

## Figures and Tables

**Table I:** Summary statistics, before multiple imputations of the missing data, of baseline and procedural characteristics in the UK TAVI dataset

Variable	Summary (% of *n* = 6676)	Missing (% of *n* = 6676)
Age, mean [range]	81.3 [29–101]	0 (0%)
Women, n (%)	3085 (46.2%)	22 (0.3%)
Weight (kg), mean [range]	74.0 [32.0–190.0]	131 (2.0%)
Height (m), mean [range]	1.6 [1.1–2.4]	159 (2.4%)
NYHA		42 (0.6%)
Class I, n (%)	185 (2.8%)	
Class II, n (%)	1116 (16.7%)	
Class III, n (%)	4186 (62.7%)	
Class IV, n (%)	1147 (17.2%)	
Creatinine, *μ*mol/L, mean [range]	114.3 [29.0–1044.0]	73 (1.1%)
Creatinine greater than 200 *μ*mol/L, n (%)	379 (5.7%)	73 (1.1%)
LVEF		59 (0.88%)
≥50%, n (%)	4074 (61.0%)	
30–49%, n (%)	1929 (28.9%)	
*<*30%, n (%)	614 (9.2%)	
Extracardiac arteriopathy, n (%)	1572 (23.5%)	88 (1.3%)
Diabetes		35 (0.52%)
Dietary control, n (%)	290 (4.3%)	
Oral medicine, n (%)	884 (13.2%)	
Insulin, n (%)	363 (5.4%)	
Dialysis, n (%)	127 (1.9%)	66 (0.99%)
MI		33 (0.49%)
Within 90 days of TAVI, n (%)	153 (2.3%)	
Within 30 days of TAVI, n (%)	65 (0.97%)	
Within 24 hours of TAVI, n (%)	6 (0.09%)	
Procedure urgency		7 (0.10%)
Elective, n (%)	5853 (87.7%)	
Urgent, n (%)	772 (11.6%)	
Emergency, n (%)	35 (0.52%)	
Salvage, n (%)	9 (0.13%)	
Valve type		31 (0.46%)
Edwards SAPIEN Valve, n (%)	3684 (55.2%)	
Medtronic CoreValve, n (%)	2735 (41.0%)	
Access route		13 (0.19%)
TF access, n (%)	4965 (74.4%)	
Transapical access, n (%)	1064 (15.9%)	
Chronic lung disease, n (%)	1879 (28.1%)	94 (1.4%)
Cerebrovascular disease, n (%)	1139 (17.1%)	35 (0.52%)
Previous cardiac surgery, n (%)	2087 (31.3%)	35 (0.52%)
Critical preoperative state, n (%)	110 (1.6%)	81 (1.2%)
PA systolic >60 mmHg	785 (11.8)	1860 (27.9%)
LMS *>*50% or Triple vessel disease, n (%)	887 (13.3%)	74 (1.1%)

*LMS*, Left main stem disease; *MI*, myocardial infarction; *NYHA*, New York Heart Association Functional Classification; *PA*, pulmonary artery; *TF*, transfemoral access route.

**Table II:** Absolute and relative differences of the expected to observed 30-day mortalities

Risk model	Expected 30-day mortality (%)	Absolute difference to observed mortality⁎	Relative difference to observed mortality† (%)
LES	21.9	16.5	405.6
ESII	8.1	2.7	150.0
STS	5.1	0.3	94.4
German AV	7.4	2.0	137.0
FRANCE-2	9.2	3.8	170.4
OBSERVANT	7.1	1.7	131.5
ACC TAVI	5.2	0.2	96.3

⁎ Calculated as the absolute value of expected minus observed.

† Calculated as (expected/observed) ×100.

**Table III:** Calibration, discrimination and Brier score for 30-day mortality in the whole cohort

Risk model	Calibration intercept (95% CI)⁎	Calibration slope (95% CI)	AUC (95% CI)	Brier score
LES	−1.75 (−1.86, −1.64)	0.35 (0.23, 0.48)	0.57 (0.54, 0.61)	0.093
ESII	−0.47 (−0.59, −0.36)	0.40 (0.28, 0.53)	0.59 (0.55, 0.62)	0.054
STS	**0.07 (−0.04, 0.18)**	0.56 (0.42, 0.71)	0.60 (0.57, 0.63)	0.051
German AV	−0.36 (−0.47, −0.25)	0.44 (0.32, 0.57)	0.59 (0.56, 0.62)	0.053
FRANCE-2	−0.60 (−0.71, −0.49)	0.69 (0.53, 0.86)	0.62 (0.59, 0.65)	0.053
OBSERVANT	−0.31 (−0.42, −0.20)	0.39 (0.25, 0.53)	0.57 (0.54, 0.60)	0.052
ACC TAVI	**0.04 (−0.07, 0.15)**	0.67 (0.52, 0.82)	0.64 (0.60, 0.67)	0.051

⁎ The reported calibration intercept is that estimated assuming a slope of one; satisfactory calibration would occur if the 95% confidence intervals for the calibration intercept and slope span zero and one respectively. Bold items indicate that the 95% CI spans the corresponding reference value.

**Table IV:** Cut-off values and the pairwise κ values for the surgical and TAVI based CPMs

CPM	Low risk⁎	High risk⁎	Fleiss's *κ*†			
Surgical based			LES	ESII	STS	German AV
LES	≤14%	>24%	n/a	0.50	0.29	0.34
ESII	≤4%	>8%	0.50	n/a	0.34	0.27
STS	≤3%	>5%	0.29	0.34	n/a	0.47
German AV‡	≤4%	>8%	0.34	0.27	0.47	n/a
TAVI based			German AV	FRANCE-2	OBSERVANT	ACC
German AV‡	≤4%	>8%	n/a	0.17	0.13	0.26
FRANCE-2	≤6%	>10%	0.17	n/a	0.14	0.33
OBSERVANT	≤4.5%	>9%	0.13	0.14	n/a	0.18
ACC	≤3%	>5%	0.26	0.33	0.18	n/a

⁎ All cut-off values were chosen to give approximately equal numbers of patients in low-, medium- and high-risk categories. Patients with predicted risks between the low- and high-risk cut-off values were classified as medium risk.

† Values give the pairwise agreement between the two indicated CPMs.

‡ The German AV model was derived in a cohort with both surgical and TAVI patients and, thus, is considered in both groups of models.

## Appendix Supplementary data

Supplementary Methods, Tables and Statistical Code used for CPM calculations. Supplementary materials
