# Supplementary material for: Inadequacy of existing clinical prediction models for predicting mortality after transcatheter aortic valve implantation
Source: Am Heart J. 2017 Feb;184:97–105. doi: 10.1016/j.ahj.2016.10.020 (PMC5333927; doi:10.1016/j.ahj.2016.10.020)
Supplement: Supplementary Methods, Tables and Statistical Code used for CPM calculations. [file mmc1.docx]

Supplementary Material

Inadequacy of Existing Clinical Prediction Models for Predicting Mortality after Transcatheter Aortic Valve Implantation

Glen P. Martin, MSc; Matthew Sperrin, PhD; Peter F. Ludman, MA, MD, FRCP, FESC; Mark A. de Belder, MA, MD, FRCP; Chris P. Gale, PhD, FRCP, FESC; William D. Toff, MD, FRCP, FESC; Neil E. Moat, MBBS, MS; Uday Trivedi, MBBS; Iain Buchan, MD, FFPH; Mamas A. Mamas, MA, DPhil, FRCP

# Supplementary Methods

## Multiple Imputation

Multiple imputation generates several complete datasets by iteratively using observed variables to impute missing values from a predictive distribution. The procedure assumes that the missing data are only related to other observed variables; that is, the missing at random (MAR) assumption. Each of the imputed datasets are analysed separately and parameter estimates from each imputed dataset are pooled by taking the average. Pooled standard errors account for both the between- and within-imputation standard error (1). Mathematical details of the imputation scheme have been published previously (1,2). In the current study, we generated ten imputed datasets using multiple imputation by chained equations (MICE), with the correspondingly named R package (3). The imputation model for each variable included both the outcome (30-day mortality) and the majority of other variables in the UK TAVI registry. Including as many predictors as possible in the imputation models makes the MAR assumption increasingly plausible. Moreover, including the outcome in the imputation models avoids underestimation of covariate-outcome associations (4). Prior to analysis, we checked all the imputations to ensure convergence of the MICE algorithms and checked that the distribution of observed and imputed values were similar. The predicted risk for each patient implied by each clinical prediction model was calculated across the ten imputed datasets. All analyses were undertaken in each dataset separately, before pooling results according to Rubin’s rules (1).

# Supplementary Results

## Supplementary Table 1: Variable translation between the LES and the 2007-2014 UK TAVI registry

| **LES Variable** | **UK TAVI Registry Field** | **Mapped TAVI Values** | **Notes** |
| --- | --- | --- | --- |
| Age | Age | Age |  |
| Female | 1.07 Sex | Female |  |
| Serum creatinine>200umol/L | 3.03 Creatinine | Any creatinine > 200 µmol/l |  |
| Extracardiac arteriopathy | 3.09 Extracardiac Arteriopathy | Yes |  |
| Pulmonary disease | 3.06 History Of Pulmonary Disease | COAD/emphysema  Asthma  Other significant pulmonary disease |  |
| Neurological dysfunction | 3.08 History Neurological Disease | CVA with residual deficit |  |
| Previous cardiac surgery | 4.01 Previous Cardiac Surgery | Previous CABG  Previous valve operation  Other operation requiring opening of the pericardium |  |
| Recent myocardial infarct | 3.05 Previous MI and interval between procedure and last MI | MI < 6 hours  MI 6-24 hours  MI 1-30 days  MI 31-90 days |  |
| LVEF 30-50% | 6.08 LV Function | 2. Fair (LVEF = 30-49%) |  |
| LVEF <30% | 6.08 LV Function | 3. Poor (LVEF <30%) |  |
| Systolic pulmonary artery pressure>60mmHg | 6.01 PA Systolic > 60mmHg | Yes |  |
| Active endocarditis | N/A | Always 0 | Assume no for all |
| Unstable angina | 5.04 CCS angina status Pre-procedure stable only & 7.06 Procedure Urgency | CCS class 3 or 4 AND urgent/emergency procedure |  |
| Emergency operation | 7.06 Procedure Urgency | 3. Emergency  4. Salvage |  |
| Critical preoperative state | 5.031 Critical Pre-Operative Status | Yes |  |
| Venticular septal rupture | N/A | Always 0 | Assume no for all |
| Other than isolated coronary surgery | N/A | Always 0.5420364 | Yes for all patients |
| Thoracic aortic surgery | N/A | Always 0 | Assume no for all |

## Supplementary Table 2: Variable translation between the ESII and the 2007-2014 UK TAVI registry

| **ESII Variable** | **UK TAVI Registry Field** | **Mapped TAVI Values** | **Notes** |
| --- | --- | --- | --- |
| Age | Age | Age |  |
| Female | 1.07 Sex | Female |  |
| Renal Impairment (Creatinine Clearance) | Age, 5.02 Weight, 1.07 Sex, 3.03 Creatinine | Creatinine Clearance calculated by the Cockcroft-Gault formula. |  |
| Dialysis (regardless of Creatinine Clearance) | 3.041 On Dialysis | Yes |  |
| Extracardiac arteriopathy | 3.09 Extracardiac Arteriopathy | Yes |  |
| Poor Mobility | 3.08 History Neurological Disease (for 2007-2012 procedures)  3.091 Poor Mobility (for 2013-2014 procedures) | CVA with residual deficit  Yes | 3.08 was used as surrogate if procedure was between 2007 and 2012 |
| Previous Cardiac Surgery | 4.01 Previous Cardiac Surgery | Previous CABG  Previous valve operation  Other operation requiring opening of the pericardium |  |
| Chronic Lung Disease | 3.06 History of Pulmonary Disease | COAD/emphysema  Asthma  Other significant pulmonary disease |  |
| Active endocarditis | N/A | Always 0 | Assume no for all |
| Critical preoperative state | 5.031 Critical Pre-Operative Status | Yes |  |
| Diabetes on insulin | 3.01 Diabetes | Diabetes (insulin) |  |
| NYHA II | 5.05 NYHA dyspnoea status | Slight limitation of ordinary physical activity |  |
| NYHA III | 5.05 NYHA dyspnoea status | Marked limitation of ordinary physical activity |  |
| NYHA IV | 5.05 NYHA dyspnoea status | Symptoms at rest or minimal activity |  |
| CCS class 4 angina | 5.04 CCS Angina Status | Symptoms at rest or minimal activity |  |
| LVEF 31-50% | 6.08 LVEF function | Fair (LVEF = 30-49%) |  |
| LVEF 21-30% | 6.08 LVEF function | Poor (LVEF <30%) |  |
| LVEF <20% | 6.08 LVEF function | N/A | Assume all patients with an LVEF < 30% had an LVEF > 20%. |
| Recent MI | 3.05 Previous MI and interval between procedure and last MI | MI < 6 hours  MI 6-24 hours  MI 1-30 days  MI 31-90 days |  |
| Moderate PA systolic pressure | 6.01 PA Systolic > 60mmHg | 0. No | Assume that any patient who has a PA Systolic of < 60mmHg lies between 31-55mmHg |
| Severe PA systolic pressure | 6.01 PA Systolic > 60mmHg | 1. Yes |  |
| Urgent | 7.06 Procedure Urgency | Urgent |  |
| Emergency | 7.06 Procedure Urgency | Emergency |  |
| Salvage | 7.06 Procedure Urgency | Salvage |  |
| Weight of the Intervention | N/A | N/A | Assume single non CABG for all cases |
| Surgery on thoracic aorta | N/A | Always 0 | Assume no for all |

## Supplementary Table 3: Variable translation between the STS and the 2007-2014 UK TAVI registry

| **STS Variable** | **UK TAVI Registry Field** | **Mapped TAVI Values** | **Notes** |
| --- | --- | --- | --- |
| Atrial Fibrillation | 3.11 Pre-Operative Heart Rhythm | Atrial fibrillation/flutter |  |
| Age | Age | Age |  |
| Body Surface Area | Height and Weight | Body Surface Area calculated by the DuBois Method |  |
| Congestive Heart Failure but no NYHA IV | N/A | Always 0 | Can only use NYHA IV as a surrogate |
| Congestive Heart Failure AND NYHA IV | 5.05 NYHA Dyspnoea Status | Symptoms at rest or minimal activity |  |
| Chronic Lung Disease | 3.06 History Of Pulmonary Disease | COAD/emphysema  Asthma  Other significant pulmonary disease | If yes, then assume the moderate category. |
| Creatinine | 3.03 Creatinine | 3.03 Creatinine µmol/l / 88.4 | Convert to mg/dL |
| Cerebrovascular Accident with prior CVA | 3.08 History of Neurological Disease | CVA with full recovery  CVA with residual deficit |  |
| Cerebrovascular Accident without prior CVA | 3.08 History of Neurological Disease | TIA or RIND |  |
| Diabetes (insulin) | 3.01 Diabetes | Diabetes (insulin) |  |
| Diabetes (noninsulin) | 3.01 Diabetes | Diabetes (dietary control)  Diabetes (oral medicine) |  |
| Dialysis | 3.041 On Dialysis | Yes |  |
| Ejection Fraction | 6.08 LV Function | Good (LVEF>50%) then Ejection Fraction = 60  Fair (LVEF 30 to 49%) then Ejection Fraction = 45  Poor (LVEF<30%) then Ejection Fraction =30 | If none of these options then Ejection fraction=50 |
| Active Endocarditis | N/A | Always 0 | Assume no for all |
| Female | 1.07 Sex | Female |  |
| Hypertension | N/A | N/A | Assume no for all |
| IABP or Inotropes | 5.031 Critical Pre-Operative Status | Yes |  |
| Immunosuppressive treatment | N/A | N/A | Assume no for all |
| Insufficiency mitral | N/A | N/A | Assume no for all |
| Insufficiency tricuspid | N/A | N/A | Assume no for all |
| Left Main Disease | 6.10 Left Main Stem Disease | LMS >50% diameter stenosis |  |
| MI<21 days | 3.05 Previous MI and interval between procedure and last MI | MI < 6 hours  MI 6-24 hours  MI 1-30 days | Assume that 1-30 days is the same as 1-21 days |
| Mitral Valve Replacement | N/A | N/A | Assume no mitral valve replacement |
| Mitral Valve Repair | N/A | N/A | Assume no mitral valve repair |
| Number of diseased vessels | 6.09 Extent of Coronary Vessel Disease | Two vessels with >50% diameter stenosis then 1  Three vessels with >50% diameter stenosis then 2 |  |
| Peripheral Vascular Disease | 3.09 Extracardiac Arteriopathy | Yes |  |
| Re-op, 1 previous operation | 4.01 Previous Cardiac Surgery | Previous CABG  Previous valve operation  Other operation requiring opening of the pericardium |  |
| Re-op, 2 or more previous operations | N/A | Always 0 | Assume no patient had more than 2 previous operations |
| Shock | 5.031 Critical Pre-Operative Status | Yes |  |
| Urgent | 7.06 Procedure Urgency | Urgent |  |
| Emergency | 7.06 Procedure Urgency | Emergency |  |
| Salvage | 7.06 Procedure Urgency | Salvage |  |
| Stenosis aortic | N/A | Always 1 |  |
| Stenosis mitral | N/A | Always 0 | Assume no for all |
| Unstable Angina | 5.04 CCS angina status Pre-procedure stable only & 7.06 Procedure Urgency | CCS class 3 or 4 AND urgent/emergency procedure |  |

## Supplementary Table 4: Variable translation between the German AV model and the 2007-2014 UK TAVI registry

| **German AV Variable** | **UK TAVI Registry Field** | **Mapped TAVI Values** | **Notes** |
| --- | --- | --- | --- |
| Age | Age | Age split into categories as per model |  |
| Female | 1.07 Sex | Female |  |
| BMI | Weight and Height | ((Weight)/(Height^2)) split into categories as per model |  |
| NYHA IV | 5.05 NYHA Dyspnoea Status | Symptoms at rest or minimal activity |  |
| MI within 3 weeks | 3.05 Previous MI and interval between procedure and last MI | MI < 6 hours  MI 6-24 hours  MI 1-30 days | Assume that 30 days is same as 3 weeks |
| Critical preoperative state | 5.031 Critical Pre-Operative Status | Yes |  |
| Pulmonary hypertension | 6.01 PA Systolic > 60mmHg | 1. Yes |  |
| No sinus rhythm | 3.11 Pre-Operative Heart Rhythm | Any option not including sinus rhythm |  |
| LVEF 30-50% | 6.08 LV function | Fair (LVEF = 30-49%) |  |
| LVEF < 30% | 6.08 LV function | Poor (LVEF <30%) |  |
| Endocarditis | N/A | N/A | Assume no for all |
| Previous Heart or aortic surgery | 4.01 Previous Cardiac surgery | Previous CABG  Previous valve operation  Other operation requiring opening of the pericardium |  |
| Arterial Vessel Disease | 3.09 Extracardiac Arteriopathy | Yes |  |
| Chronic obstructive pulmonary disease | 3.08 History of Pulmonary Disease | COAD/emphysema  Asthma  Other significant pulmonary disease |  |
| Pre-op Dialysis or pre-op renal fail | 3.041 On Dialysis and 3.03 Creatinine Renal Function | 3.041: Yes  3.03: Creatinine > 200 µmol/l |  |
| Emergency operation | 7.06 Procedure Urgency | Emergency or salvage |  |

## Supplementary Table 5: Variable translation between the FRANCE-2 model and the 2007-2014 UK TAVI registry

| **FRANCE-2 Variable** | **UK TAVI Registry Field** | **Mapped TAVI Values** | **Notes** |
| --- | --- | --- | --- |
| Age | Age | Age split into categories as per model |  |
| BMI | Weight and Height | ((Weight)/(Height^2)) split into categories as per model |  |
| Respiratory Insufficiency | 3.06 History of pulmonary disease | COAD/emphysema  Asthma  Other significant pulmonary disease |  |
| Acute pulmonary oedema | N/A | N/A | Assume no for all |
| NYHA Class IV | 5.05 NYHA Dyspnoea Status | Symptoms at rest or minimal activity |  |
| Dialysis | 3.041 On Dialysis | Yes |  |
| Pulmonary hypertension | 6.01 PA Systolic > 60mmHg | Yes |  |
| Critical preoperative state | 5.031 Critical Pre-Operative Status | Yes |  |
| Transapical Access | 7.10 Delivery Approach | Transapical |  |
| Other Access | 7.10 Delivery Approach | Any option other than Transapical or Transfemoral access |  |

## Supplementary Table 6: Variable translation between the OBSERVANT model and the 2007-2014 UK TAVI registry

| **OBSERVANT Variable** | **UK TAVI Registry Field** | **Mapped TAVI Values** | **Notes** |
| --- | --- | --- | --- |
| GFR<45 mL/min | Age, 5.02 Weight, 1.07 Sex, 3.03 Creatinine | Calculated by the Modification of Diet in Renal Disease formula |  |
| Critical preoperative state | 5.031 Critical Pre-Operative Status | Yes |  |
| Pulmonary hypertension | 6.01 PA Systolic > 60mmHg | Yes | Pulmonary hypertension |
| Diabetes | 3.01 Diabetes | Diabetes (dietary control)  Diabetes (oral medicine)  Diabetes (insulin) |  |
| NYHA Class IV | 5.05 NYHA Dyspnoea Status | Symptoms at rest or minimal activity |  |
| Prior BAV | 4.021 Balloon aortic valvuloplasty prior to date of TAVI  7.074 Aortic balloon valvuloplasty before valve deployment | Yes  Completed |  |
| LVEF<40% | 6.08 LV function | Fair (LVEF = 30-49%)  Poor (LVEF <30%) | assume <50% is same as <40% |

## Supplementary Table 7: Variable translation between the ACC TAVI model and the 2007-2014 UK TAVI registry

| **ACC Risk Model Variable** | **UK TAVI Registry Field** | **Mapped TAVI Values** | **Notes** |
| --- | --- | --- | --- |
| Age per 5-year increments | Age at Op | Age divided by 5 rounded down to whole number |  |
| Glomerular filtration rate per 5-U increments | Age at Op, 1.07 Sex, 1.08 Ethnic origin, 3.03 Creatinine | Calculated by the Modification of Diet in Renal Disease formula |  |
| Dialysis vs no dialysis | 3.041 On dialysis | Yes |  |
| NYHA class IV | 5.05 NYHA dyspnoea status | Symptoms at rest or minimal activity |  |
| Severe chronic lung disease | 3.06 History of pulmonary disease | COAD/emphysema  Asthma  Other significant pulmonary disease |  |
| Nonfemoral access site | 7.10 Delivery approach | **Not**:  “Femoral – percutaneous” or  “Femoral – surgical” |  |
| Acuity category: |  |  |  |
| 2 | 5.031 Critical Pre-Operative Status  3.05 Previous MI  7.06 Procedure Urgency | Yes if  Procedure urgency = “Urgent”  AND  Critical pre-operative status = “No”  AND  Previous MI is No MI |  |
| 3 | 5.031 Critical Pre-Operative Status  3.05 Previous MI  7.06 Procedure Urgency | Yes if  Procedure urgency = “Urgent”/ “Elective”  AND  Critical pre-operative status = “Yes”  AND  Previous MI is not recorded as 6-24 hours or <6 hours |  |
| 4 | 5.031 Critical Pre-Operative Status  3.05 Previous MI  7.06 Procedure Urgency | Yes if  Procedure urgency = “Emergency”/ “Salvage”  OR  Previous MI = MI 6-24 hours or MI <6 hours |  |

## Supplementary Table 8: Observed and Expected 30-day mortalities over strata.

| **Strata (number of patients)** | **Observed 30-day mortality** | **LES** | **ESII** | **STS** | **German AV** | **FRANCE-2 TAVI Model** | **OBSERVANT TAVI Model** | **ACC TAVI Model** |
| --- | --- | --- | --- | --- | --- | --- | --- | --- |
| Whole Cohort (6676) | 5.4% | 21.9% | 8.1% | 5.1% | 7.4% | 9.2% | 7.1% | 5.2% |
| Age ≤ 75 years (1264) | 4.7% | 16.1% | 6.9% | 3.4% | 3.3% | 9.8% | 7.4% | 4.6% |
| Age > 75 years (5412) | 5.5% | 23.2% | 8.4% | 5.4% | 8.4% | 9.1% | 7.0% | 5.3% |
| Male (3579-3583) | 5.2% | 22.4% | 8.5% | 4.6% | 6.8% | 9.4% | 7.2% | 5.2% |
| Female (3093-3097) | 5.7% | 21.2% | 7.7% | 5.5% | 8.2% | 9.0% | 7.0% | 5.2% |
| Diabetic (1540-1550) | 4.9% | 22.6% | 9.2% | 6.0% | 7.2% | 8.9% | 11.5% | 5.8% |
| Non-diabetic (5126-5136) | 5.5% | 21.6% | 7.8% | 4.8% | 7.5% | 9.3% | 5.8% | 5.1% |
| TF (4974-4977) | 4.3% | 20.9% | 7.7% | 5.0% | 7.3% | 7.5% | 7.1% | 4.2% |
| Non-TF (1699-1702) | 8.6% | 24.5% | 9.5% | 5.4% | 7.7% | 14.3% | 7.0% | 8.0% |
| SAPIEN (3700-3706) | 5.8% | 21.9% | 8.3% | 5.2% | 7.6% | 9.8% | 6.9% | 5.6% |
| CoreValve (2742-2748) | 5.1% | 21.9% | 8.1% | 4.9% | 7.3% | 8.6% | 7.4% | 4.8% |
| Prior CABG (1733-1744) | 5.3% | 30.0% | 13.1% | 5.1% | 6.9% | 8.8% | 7.4% | 4.9% |
| No Prior CABG (4932-4943) | 5.4% | 19.0% | 6.4% | 5.0% | 7.6% | 9.4% | 7.0% | 5.3% |
| LVEF<50% (2562-2573) | 6.6% | 29.1% | 11.4% | 5.9% | 9.6% | 10.2% | 10.0% | 5.7% |
| LVEF≥50% (4103-4114) | 4.7% | 17.3% | 6.1% | 4.5% | 6.1% | 8.6% | 5.3% | 4.9% |
| Elective (5856-5859) | 5.0% | 20.9% | 7.2% | 4.5% | 6.9% | 8.8% | 6.6% | 4.7% |
| Non-Elective (817-820) | 8.4% | 28.6% | 14.9% | 9.4% | 11.4% | 12.3% | 10.6% | 8.7% |

*A range is given for the number of patients in each subgroup for multiple imputed datasets. CABG = Coronary Artery Bypass Graft, LVEF = Left Ventricular Ejection Fraction, TF = Transfemoral Access Route.*

**Supplementary Table 9. Calibration, discrimination and Brier score for 30-day mortality in the whole cohort from the sensitivity analysis that excluded procedures conducted in 2007 and 2008.**

| **Risk Model** | **Calibration Intercept (95% CI) *** | **Calibration Slope (95% CI)** | **AUC (95% CI)** | **Brier Score** |
| --- | --- | --- | --- | --- |
| LES | -1.80 (-1.91, -1.68) | 0.37 (0.24, 0.50) | 0.58 (0.54, 0.61) | 0.091 |
| ESII | -0.52 (-0.64, -0.41) | 0.41 (0.28, 0.54) | 0.59 (0.55, 0.62) | 0.052 |
| STS | **0.02 (-0.09, 0.14)** | 0.58 (0.43, 0.73) | 0.60 (0.57, 0.63) | 0.049 |
| German AV | -0.41 (-0.53, -0.30) | 0.48 (0.35, 0.61) | 0.60 (0.57, 0.64) | 0.050 |
| FRANCE-2 | -0.65 (-0.76, -0.54) | 0.71 (0.53, 0.88) | 0.63 (0.60, 0.66) | 0.051 |
| OBSERVANT | -0.36 (-0.47, -0.24) | 0.35 (0.21, 0.50) | 0.56 (0.53, 0.59) | 0.051 |
| ACC TAVI | **-0.01 (-0.12, 0.10)** | 0.69 (0.53, 0.85) | 0.64 (0.61, 0.67) | 0.049 |

**The reported calibration intercept is that estimated assuming a slope of one; satisfactory calibration would occur if the 95% confidence intervals for the calibration intercept and slope span zero and one respectively.*

## Supplementary Table 10: Calibration and discrimination analysis for 30-day mortality over all subgroups considered.

| **Group/Strata** | **Risk Model** | **Calibration Intercept (95% CI)** | **Calibration Slope (95% CI)** | **AUC (95% CI)** | **Brier Score** |
| --- | --- | --- | --- | --- | --- |
| Age ≤ 75 years | LES | -1.48 (-1.75, -1.21) | 0.19 (-0.09, 0.46) | 0.55 (0.47, 0.64) | 0.072 |
|  | ESII | -0.42 (-0.69, -0.15) | 0.29 (0.03, 0.56) | 0.58 (0.50, 0.66) | 0.049 |
|  | STS | 0.35 (0.08, 0.61) | 0.37 (0.04, 0.71) | 0.59 (0.52, 0.66) | 0.046 |
|  | German AV | 0.39 (0.13, 0.66) | 0.34 (0.02, 0.66) | 0.58 (0.51, 0.66) | 0.046 |
|  | FRANCE2 TAVI | -0.81 (-1.08, -0.55) | 0.39 (0.02, 0.76) | 0.59 (0.53, 0.66) | 0.051 |
|  | OBSERVANT | -0.50 (-0.77, -0.24) | 0.35 (0.03, 0.67) | 0.57 (0.49, 0.65) | 0.048 |
|  | ACC TAVI | **0.04 (-0.23, 0.31)** | 0.30 (-0.02, 0.62) | 0.59 (0.51, 0.66) | 0.047 |
| Age > 75 years | LES | -1.79 (-1.92, -1.67) | 0.40 (0.26, 0.54) | 0.58 (0.54, 0.61) | 0.098 |
|  | ESII | -0.48 (-0.61, -0.36) | 0.43 (0.29, 0.57) | 0.59 (0.55, 0.62) | 0.055 |
|  | STS | **0.02 (-0.10, 0.14)** | 0.63 (0.47, 0.80) | 0.60 (0.57, 0.64) | 0.052 |
|  | German AV | -0.47 (-0.59, -0.35) | 0.54 (0.39, 0.69) | 0.60 (0.57, 0.63) | 0.054 |
|  | FRANCE2 TAVI | -0.55 (-0.67, -0.43) | 0.78 (0.59, 0.97) | 0.63 (0.60, 0.66) | 0.054 |
|  | OBSERVANT | -0.27 (-0.39, -0.15) | 0.40 (0.25, 0.56) | 0.57 (0.53, 0.60) | 0.054 |
|  | ACC TAVI | **0.04 (-0.08, 0.16)** | 0.79 (0.62, 0.97) | 0.65 (0.61, 0.68) | 0.052 |
| Male | LES | -1.84 (-2.00, -1.69) | 0.46 (0.30, 0.62) | 0.61 (0.56, 0.65) | 0.094 |
|  | ESII | -0.58 (-0.73, -0.42) | 0.47 (0.31, 0.63) | 0.61 (0.57, 0.66) | 0.052 |
|  | STS | **0.12 (-0.03, 0.27)** | 0.59 (0.39, 0.78) | 0.61 (0.57, 0.65) | 0.049 |
|  | German AV | -0.30 (-0.46, -0.15) | 0.53 (0.36, 0.69) | 0.62 (0.57, 0.66) | 0.050 |
|  | FRANCE2 TAVI | -0.67 (-0.82, -0.52) | 0.72 (0.50, 0.95) | 0.62 (0.58, 0.67) | 0.051 |
|  | OBSERVANT | -0.38 (-0.53, -0.22) | 0.43 (0.23, 0.62) | 0.57 (0.52, 0.62) | 0.050 |
|  | ACC TAVI | **-0.01 (-0.16, 0.14)** | 0.60 (0.39, 0.80) | 0.62 (0.58, 0.66) | 0.049 |
| Female | LES | -1.64 (-1.80, -1.48) | 0.22 (0.02, 0.41) | 0.54 (0.49, 0.59) | 0.092 |
|  | ESII | -0.36 (-0.52, -0.19) | 0.33 (0.14, 0.52) | 0.56 (0.51, 0.60) | 0.056 |
|  | STS | **0.02 (-0.13, 0.18)** | 0.54 (0.31, 0.76) | 0.58 (0.54, 0.63) | 0.053 |
|  | German AV | -0.42 (-0.58, -0.26) | 0.32 (0.12, 0.52) | 0.56 (0.52, 0.61) | 0.056 |
|  | FRANCE2 TAVI | -0.52 (-0.68, -0.37) | 0.66 (0.42, 0.90) | 0.63 (0.58, 0.67) | 0.055 |
|  | OBSERVANT | -0.23 (-0.39, -0.08) | 0.36 (0.16, 0.56) | 0.57 (0.52, 0.61) | 0.055 |
|  | ACC TAVI | **0.10 (-0.06, 0.25)** | **0.77 (0.54, 1.00)** | 0.65 (0.61, 0.69) | 0.053 |
| Diabetic | LES | -1.89 (-2.13, -1.66) | 0.58 (0.33, 0.83) | 0.62 (0.55, 0.68) | 0.092 |
|  | ESII | -0.71 (-0.95, -0.47) | 0.67 (0.42, 0.93) | 0.64 (0.58, 0.71) | 0.050 |
|  | STS | **-0.20 (-0.44, 0.03)** | **0.83 (0.52, 1.13)** | 0.64 (0.58, 0.71) | 0.046 |
|  | German AV | **-0.42 (-0.66, 0.19)** | 0.55 (0.30, 0.81) | 0.63 (0.57, 0.70) | 0.049 |
|  | FRANCE2 TAVI | -0.66 (-0.89, -0.42) | **0.90 (0.55, 1.25)** | 0.66 (0.60, 0.72) | 0.049 |
|  | OBSERVANT | -0.95 (-1.19, -0.72) | **0.88 (0.56, 1.20)** | 0.67 (0.60, 0.73) | 0.052 |
|  | ACC TAVI | **-0.11 (-0.35, 0.12)** | **0.92 (0.60, 1.24)** | 0.70 (0.64, 0.76) | 0.046 |
| Non-diabetic | LES | -1.70 (-1.83, -1.58) | 0.29 (0.15, 0.43) | 0.56 (0.53, 0.60) | 0.093 |
|  | ESII | -0.40 (-0.53, -0.27) | 0.33 (0.19, 0.48) | 0.57 (0.54, 0.61) | 0.055 |
|  | STS | 0.16 (0.04, 0.28) | 0.51 (0.34, 0.68) | 0.59 (0.55, 0.62) | 0.052 |
|  | German AV | -0.35 (-0.47, -0.22) | 0.41 (0.26, 0.55) | 0.58 (0.55, 0.62) | 0.054 |
|  | FRANCE2 TAVI | -0.58 (-0.71, -0.46) | 0.63 (0.44, 0.82) | 0.61 (0.58, 0.65) | 0.055 |
|  | OBSERVANT | **-0.05 (-0.17, 0.07)** | 0.40 (0.23, 0.58) | 0.57 (0.53, 0.60) | 0.053 |
|  | ACC TAVI | **0.08 (-0.04, 0.21)** | 0.60 (0.43, 0.78) | 0.62 (0.58, 0.65) | 0.052 |
| TF | LES | -1.94 (-2.08, -1.79) | 0.28 (0.12, 0.44) | 0.55 (0.51, 0.59) | 0.084 |
|  | ESII | -0.66(-0.80, -0.52) | 0.39 (0.23, 0.54) | 0.57 (0.53, 0.61) | 0.045 |
|  | STS | -0.15 (-0.29, -0.01) | 0.59 (0.40, 0.77) | 0.59 (0.55, 0.63) | 0.041 |
|  | German AV | -0.60 (-0.74, -0.46) | 0.37 (0.21, 0.54) | 0.58 (0.53, 0.62) | 0.044 |
|  | FRANCE2 TAVI | -0.60 (-0.74, -0.46) | 0.71 (0.46, 0.96) | 0.59 (0.55, 0.63) | 0.042 |
|  | OBSERVANT | -0.56 (-0.70, -0.42) | 0.45 (0.28, 0.63) | 0.58 (0.53, 0.62) | 0.043 |
|  | ACC TAVI | **0.01 (-0.13, 0.15)** | 0.66 (0.44, 0.88) | 0.60 (0.55, 0.64) | 0.041 |
| Non-TF | LES | -1.38 (-1.56, -1.20) | 0.37 (0.17, 0.57) | 0.58 (0.53, 0.63) | 0.119 |
|  | ESII | **-0.11 (-0.30, 0.07)** | 0.32 (0.12, 0.53) | 0.57 (0.52, 0.62) | 0.082 |
|  | STS | 0.53 (0.36, 0.70) | 0.47 (0.23, 0.71) | 0.59 (0.54, 0.64) | 0.079 |
|  | German AV | **0.13 (-0.05, 0.31)** | 0.53 (0.33, 0.74) | 0.61 (0.57, 0.66) | 0.078 |
|  | FRANCE2 TAVI | -0.60 (-0.77, -0.43) | 0.31 (-0.02, 0.65) | 0.54 (0.49, 0.59) | 0.085 |
|  | OBSERVANT | 0.23 (0.06, 0.41) | 0.31 (0.08, 0.54) | 0.56 (0.51, 0.61) | 0.076 |
|  | ACC TAVI | **0.08 (-0.09, 0.26)** | 0.38 (0.10, 0.67) | 0.58 (0.53, 0.63) | 0.080 |
| SAPIEN | LES | -1.65 (-1.79, -1.51) | 0.35 (0.19, 0.52) | 0.58 (0.54, 0.62) | 0.095 |
|  | ESII | -0.41 (-0.55, -0.26) | 0.35 (0.19, 0.52) | 0.58 (0.54, 0.62) | 0.058 |
|  | STS | **0.12 (-0.02, 0.26)** | 0.50 (0.30, 0.69) | 0.59 (0.55, 0.63) | 0.055 |
|  | German AV | -0.30 (-0.44, -0.16) | 0.46 (0.29, 0.69) | 0.60 (0.56, 0.63) | 0.056 |
|  | FRANCE2 TAVI | -0.58 (-0.72, -0.44) | 0.67 (0.45, 0.88) | 0.62 (0.58, 0.66) | 0.057 |
|  | OBSERVANT | -0.19 (-0.33, -0.05) | 0.29 (0.10, 0.47) | 0.55 (0.51, 0.59) | 0.057 |
|  | ACC TAVI | **0.05 (-0.09, 0.19)** | 0.67 (0.47, 0.87) | 0.64 (0.60, 0.68) | 0.055 |
| CoreValve | LES | -1.83 (-2.00, -1.65) | 0.36 (0.18, 0.55) | 0.57 (0.52, 0.63) | 0.092 |
|  | ESII | -0.53 (-0.70, -0.35) | 0.47 (0.28, 0.65) | 0.59 (0.54, 0.64) | 0.051 |
|  | STS | **0.05 (-0.13, 0.22)** | 0.64 (0.41, 0.86) | 0.60 (0.55, 0.66) | 0.048 |
|  | German AV | -0.40 (-0.58, -0.22) | 0.42 (0.23, 0.62) | 0.59 (0.54, 0.64) | 0.050 |
|  | FRANCE2 TAVI | -0.57 (-0.74, -0.40) | 0.70 (0.43, 0.96) | 0.62 (0.57, 0.67) | 0.050 |
|  | OBSERVANT | -0.41 (-0.58, -0.23) | 0.57 (0.36, 0.79) | 0.60 (0.55, 0.66) | 0.049 |
|  | ACC TAVI | **0.08 (-0.09, 0.25)** | 0.64 (0.39, 0.90) | 0.62 (0.57, 0.67) | 0.048 |
| Previous CABG | LES | -2.22 (-2.43, -2.01) | 0.58 (0.33, 0.83) | 0.61 (0.55, 0.67) | 0.127 |
|  | ESII | -1.05 (-1.27, -0.84) | 0.65 (0.39, 0.93) | 0.64 (0.58, 0.69) | 0.060 |
|  | STS | **0.04 (-0.17, 0.26)** | 0.52 (0.24, 0.81) | 0.58 (0.52, 0.64) | 0.050 |
|  | German AV | -0.30 (-0.52, -0.09) | 0.48 (0.24, 0.71) | 0.59 (0.53, 0.65) | 0.051 |
|  | FRANCE2 TAVI | -0.56 (-0.78, -0.35) | **0.74 (0.42, 1.07)** | 0.63 (0.57, 0.69) | 0.052 |
|  | OBSERVANT | -0.38 (-0.59, -0.16) | 0.47 (0.20, 0.74) | 0.58 (0.52, 0.64) | 0.051 |
|  | ACC TAVI | **0.07 (-0.14, 0.29)** | 0.64 (0.35, 0.93) | 0.64 (0.58, 0.70) | 0.050 |
| No previous CABG | LES | -1.53 (-1.66, -1.40) | 0.34 (0.19, 0.49) | 0.57 (0.54, 0.61) | 0.081 |
|  | ESII | -0.18 (-0.31, -0.06) | 0.45 (0.30, 0.61) | 0.59 (0.55, 0.62) | 0.052 |
|  | STS | **0.08 (-0.04, 0.20)** | 0.58 (0.41, 0.75) | 0.60 (0.57, 0.64) | 0.051 |
|  | German AV | -0.38 (-0.51, -0.26) | 0.43 (0.28, 0.58) | 0.59 (0.56, 0.63) | 0.053 |
|  | FRANCE2 TAVI | -0.61 (-0.74, -0.49) | 0.67 (0.48, 0.87) | 0.62 (0.59, 0.66) | 0.054 |
|  | OBSERVANT | -0.29 (-0.55, -0.30) | 0.37 (0.21, 0.53) | 0.57 (0.53, 0.60) | 0.053 |
|  | ACC TAVI | **0.03 (-0.10, 0.15)** | 0.69 (0.50, 0.87) | 0.63 (0.60, 0.67) | 0.051 |
| LVEF<50% | LES | -1.96 (-2.12, -1.79) | 0.48 (0.30, 0.66) | 0.61 (0.56, 0.65) | 0.129 |
|  | ESII | -0.65 (-0.82, -0.49) | 0.52 (0.34, 0.70) | 0.62 (0.57, 0.66) | 0.067 |
|  | STS | **0.12 (-0.04, 0.28)** | 0.64 (0.44, 0.84) | 0.62 (0.57, 0.66) | 0.060 |
|  | German AV | -0.44 (-0.60, -0.28) | 0.52 (0.34, 0.70) | 0.62 (0.58, 0.67) | 0.064 |
|  | FRANCE2 TAVI | -0.50 (-0.66, -0.34) | 0.73 (0.50, 0.97) | 0.63 (0.59, 0.68) | 0.063 |
|  | OBSERVANT | -0.48 (-0.64, -0.32) | 0.51 (0.30, 0.72) | 0.60 (0.55, 0.64) | 0.064 |
|  | ACC TAVI | **0.15 (-0.01, 0.31)** | 0.69 (0.47, 0.91) | 0.65 (0.60, 0.69) | 0.061 |
| LVEF≥50% | LES | -1.54 (-1.69, -1.39) | 0.13 (-0.08, 0.33) | 0.52 (0.48, 0.57) | 0.070 |
|  | ESII | -0.30 (-0.45, -0.15) | 0.21 (0.01, 0.42) | 0.54 (0.50, 0.59) | 0.046 |
|  | STS | **0.03 (-0.12, 0.16)** | 0.41 (0.18, 0.63) | 0.57 (0.53, 0.61) | 0.045 |
|  | German AV | -0.29 (-0.44 -0.14) | 0.28 (0.09, 0.48) | 0.55 (0.51, 0.60) | 0.046 |
|  | FRANCE2 TAVI | -0.68 (-0.82, -0.53) | 0.59 (0.35, 0.83) | 0.60 (0.56, 0.64) | 0.047 |
|  | OBSERVANT | **-0.14 (-0.28, 0.01)** | 0.20 (-0.04, 0.43) | 0.52 (0.47, 0.57) | 0.045 |
|  | ACC TAVI | **-0.05 (-0.20, 0.10)** | 0.60 (0.39, 0.82) | 0.62 (0.57, 0.66) | 0.045 |
| Elective | LES | -1.75 (-1.87, -1.63) | 0.29 (0.15, 0.44) | 0.56 (0.52, 0.60) | 0.086 |
|  | ESII | -0.41 (-0.54, -0.29) | 0.37 (0.22, 0.53) | 0.58 (0.54, 0.61) | 0.049 |
|  | STS | 0.12 (0.00, 0.24) | 0.52 (0.33, 0.72) | 0.59 (0.55, 0.62) | 0.047 |
|  | German AV | -0.36 (-0.48, -0.24) | 0.38 (0.23, 0.53) | 0.58 (0.54, 0.61) | 0.049 |
|  | FRANCE2 TAVI | -0.63 (-0.75, -0.51) | 0.77 (0.58, 0.97) | 0.63 (0.60, 0.67) | 0.049 |
|  | OBSERVANT | -0.32 (-0.44, -0.20) | 0.33 (0.16, 0.49) | 0.55 (0.52, 0.59) | 0.049 |
|  | ACC TAVI | **0.06 (-0.06, 0.18)** | 0.73 (0.54, 0.91) | 0.63 (0.60, 0.67) | 0.047 |
| Non-Elective | LES | -1.74 (-2.00, -1.47) | 0.37 (0.14, 0.60) | 0.61 (0.53, 0.68) | 0.140 |
|  | ESII | -0.73 (-0.99, -0.47) | 0.33 (0.09, 0.58) | 0.58 (0.51, 0.66) | 0.090 |
|  | STS | **-0.12 (-0.38, 0.13)** | 0.53 (0.23, 0.83) | 0.61 (0.53, 0.69) | 0.076 |
|  | German AV | -0.37 (-0.63, -0.11) | 0.46 (0.22, 0.71) | 0.63 (0.55, 0.70) | 0.081 |
|  | FRANCE2 TAVI | -0.45 (-0.70, -0.11) | 0.29 (-0.05, 0.62) | 0.54 (0.47, 0.62) | 0.083 |
|  | OBSERVANT | -0.27 (-0.53, -0.02) | 0.41 (0.12, 0.69) | 0.59 (0.51, 0.67) | 0.080 |
|  | ACC TAVI | **-0.03 (-0.28, 0.22)** | 0.32 (-0.04, 0.68) | 0.58 (0.51, 0.66) | 0.079 |

*The reported calibration intercept is that estimated assuming a slope of one; satisfactory calibration would occur if the 95% confidence intervals for the calibration intercept and slope span zero and one respectively. Bold items represent significantly acceptable performance. CABG: Coronary Artery Bypass Graft, LVEF: Left Ventricular Ejection Fraction, TF: Transfemoral Access route.*

**Supplementary Table 11. Cut-off values and the pairwise kappa values for the surgical and TAVI based CPMs for the sensitivity analysis that stratified in a 1:3:1 ratio.**

| **CPM** | **Low Risk*** | **High Risk*** | **Fleiss’s kappa** *†* | | | |
| --- | --- | --- | --- | --- | --- | --- |
| Surgical Based |  |  | LES | ESII | STS | German AV |
| LES | ≤ 11% | >31% | n/a | 0.52 | 0.34 | 0.39 |
| ESII | ≤ 3% | >12% | 0.52 | n/a | 0.36 | 0.28 |
| STS | ≤ 2.5% | >7% | 0.34 | 0.36 | n/a | 0.52 |
| German AV^‡^ | ≤ 3% | >11% | 0.39 | 0.28 | 0.52 | n/a |
| TAVI Based |  |  | German AV | FRANCE-2 | OBSERVANT | ACC |
| German AV^‡^ | ≤ 3% | >11% | n/a | 0.14 | 0.15 | 0.28 |
| FRANCE-2 | ≤ 5% | >11% | 0.14 | n/a | 0.17 | 0.33 |
| OBSERVANT | ≤ 3% | >10% | 0.15 | 0.17 | n/a | 0.15 |
| ACC | ≤ 2.5% | >7% | 0.28 | 0.33 | 0.15 | n/a |

**: All cut-off values were chosen to give sample sizes across the low-, medium- and high-risk groups at a ratio of approximately 1:3:1. †:Values give the pairwise agreement between the two indicated CPMs. ‡: The German AV model was derived in a cohort with both surgical and TAVI patients, thus is considered in both groups of models.*

# Supplementary References

1. Rubin DB. Multiple Imputation for Nonresponse in Surveys. John Wiley & Sons; 1987.

2. Little R. Regression with missing X’s: a review. J Am Stat Assoc. 1992;87(420):1227–37.

3. Buuren S Van, Groothuis-Oudshoorn K. mice: Multivariate Imputation by Chained Equations in R. J Stat Softw. 2011;45(3):1–67.

4. Sterne JAC, White IR, Carlin JB, Spratt M, Royston P, Kenward MG, et al. Multiple imputation for missing data in epidemiological and clinical research: potential and pitfalls. BMJ. 2009;338(1):b2393–b2393.

# CPM Calculator R Code

## Logistic EuroSCORE R Code

Logistic.EuroSCORE <- function(data){
 age.x <- ifelse(data$Age.at.Op<=59, 1, (data$Age.at.Op-58)) #If age is less than or equal to 59 then 1, otherwise increases by one point for each year above 59 (i.e Age 60=2, age 61=3, etc.)

 gender.x <- ifelse(data$X1.07.Sex=="Female", 1, 0) #If female then one otherwise zero.

 Creat.x <- ifelse(data$X3.03.Creatinine>=200, 1, 0) #If the creat is above 200 or on diaylsis then 1.

 Extracardiac.Arteriopathy.x <- ifelse(data$X3.09.Extracardiac.arteriopathy=="Yes" ,1, 0)

 Pulmonary.Dis.x <- ifelse(data$X3.06.History.of.pulmonary.disease=="COAD/emphysema" | data$X3.06.History.of.pulmonary.disease=="Asthma" | data$X3.06.History.of.pulmonary.disease=="Other significant pulmonary disease", 1, 0) #.

 Neuro.dys.x <- ifelse(data$X3.08.History.of.neurological.disease=="CVA with residual deficit", 1, 0)

 Previous.cardiac.surg.x <- ifelse(data$X4.01.1.Previous.CABG=="Yes" |data$X4.01.2.Previous.Valve.Op=="Yes" |data$X4.01.3.Other.op.requiring.opening.of.pericardium=="Yes", 1, 0)

 MI.x <- ifelse(data$X3.05.Previous.MI.and.interval.between.procedure.and.last.MI=="No previous MI" | data$X3.05.Previous.MI.and.interval.between.procedure.and.last.MI=="MI > 90 days", 0, 1)

 LV.Fair.x <- ifelse(data$X6.08.LV.function=="Fair (LVEF = 30-49%)", 1, 0)

 LV.Poor.x <- ifelse(data$X6.08.LV.function=="Poor (LVEF <30%)", 1, 0)

 Systolic.pulmonary.x <- ifelse(data$X6.01.PA.systolic...60mmHg=="Yes", 1, 0)

 Active.endocarditis.X <- rep(0, dim(data)[1]) #assume no for all.

 CCS34 <- ifelse(data$X5.04.CCS.angina.status..Pre.procedure..stable.only.=="Marked limitation of ordinary physical activity" | data$X5.04.CCS.angina.status..Pre.procedure..stable.only.=="Symptoms at rest or minimal activity", 1, 0)
 Urgent <- ifelse(data$X7.06.Procedure.urgency=="Urgent" | data$X7.06.Procedure.urgency=="Emergency", 1, 0)
 Unstable.angina.x <- ifelse(CCS34==1 & Urgent==1, 1, 0)
 rm(CCS34); rm(Urgent)

 Urgency.x <- ifelse(data$X7.06.Procedure.urgency=="Emergency" | data$X7.06.Procedure.urgency=="Salvage", 1, 0)

 Critical.preop.state.x <- ifelse(data$X5.031.Critical.pre.operative.status..v4.=="Yes", 1, 0)

 Venticular.septal.rupture <- rep(0, dim(data)[1]) #assume no for all.

 other.than.isolated.x <- rep(1, dim(data)[1]) #Yes for all patients.

 Thoracic.aortic.surgery.x <- rep(0, dim(data)[1]) #assume no for all.

 design.mat <- matrix(c(rep(1, dim(data)[1]), age.x, gender.x, Creat.x, Extracardiac.Arteriopathy.x, Pulmonary.Dis.x, Neuro.dys.x, Previous.cardiac.surg.x, MI.x, LV.Fair.x, LV.Poor.x, Systolic.pulmonary.x, Active.endocarditis.X, Unstable.angina.x, Urgency.x, Critical.preop.state.x, Venticular.septal.rupture, other.than.isolated.x, Thoracic.aortic.surgery.x), ncol=19, byrow=FALSE)#.

 coeffs <- c(-4.789594, 0.0666354, 0.3304052, 0.6521653, 0.6558917, 0.4931341, 0.841626, 1.002625, 0.5460218, 0.4191643, 1.094443, 0.7676924, 1.101265, 0.5677075, 0.7127953, 0.9058132, 1.462009, 0.5420364, 1.159787)

 LP <- design.mat%*%coeffs
 LES <- (exp(LP)/(1+exp(LP)))*100
 return(LES)
}

## EuroSCORE II R Code

EuroSCOREII <- function(data){
 # Cockroft-Gault creatinine clearance formula.
 Creatine.clearance <- ifelse(data$X1.07.Sex=="Female",
 ((140-data$Age.at.Op)*(data$X5.02.Weight)*(0.85))/(72*(data$X3.03.Creatinine/88.4)),
 ((140-data$Age.at.Op)*(data$X5.02.Weight))/(72*(data$X3.03.Creatinine/88.4))) #mL/min.

 age.x <- ifelse(data$Age.at.Op<=60, 1, (data$Age.at.Op-59)) #If age is less than or equal to 60 then 1, otherwise increases by one point for each year above 60 (i.e Age 61=2, age 62=3, etc.).

 gender.x <- ifelse(data$X1.07.Sex=="Female", 1, 0)

 renal.impair.dialysis.x <- ifelse(data$X3.041.On.dialysis=="Yes",1, 0)

 renal.impair.fair.x <- ifelse(Creatine.clearance>50 & Creatine.clearance<85 & renal.impair.dialysis.x!=1, 1, 0)

 renal.impair.poor.x <- ifelse(Creatine.clearance<=50 & renal.impair.dialysis.x!=1, 1, 0)

 Extra.arterio.x <- ifelse(data$X3.09.Extracardiac.arteriopathy=="Yes", 1, 0)

 poor.mobility.x <- ifelse(data$X7.01.Date.and.time.of.operation_year!="2013" & data$X7.01.Date.and.time.of.operation_year!="2014",ifelse(data$X3.08.History.of.neurological.disease=="CVA with residual deficit", 1, 0), ifelse(data$X3.091.Poor.mobility=="Yes", 1, 0))

 pre.surgery.x <- ifelse(data$X4.01.1.Previous.CABG=="Yes" |data$X4.01.2.Previous.Valve.Op=="Yes" |data$X4.01.3.Other.op.requiring.opening.of.pericardium=="Yes", 1, 0)

 chronic.lung.x <- ifelse(data$X3.06.History.of.pulmonary.disease=="COAD/emphysema" |data$X3.06.History.of.pulmonary.disease=="Asthma" |data$X3.06.History.of.pulmonary.disease=="Other significant pulmonary disease", 1, 0)

 Active.endocarditis.X <- rep(0, dim(data)[1]) #assume no for all.

 critical.preop.x <- ifelse(data$X5.031.Critical.pre.operative.status..v4.=="Yes", 1, 0)

 diabetes.insulin.x <- ifelse(data$X3.01.Diabetes=="Diabetes (insulin)", 1, 0)

 NYHA2.x <- ifelse(data$X5.05.NYHA.dyspnoea.status..Pre.procedure..stable.only.=="Slight limitation of ordinary physical activity", 1, 0)
 NYHA3.x <- ifelse(data$X5.05.NYHA.dyspnoea.status..Pre.procedure..stable.only.=="Marked limitation of ordinary physical activity", 1, 0)
 NYHA4.x <- ifelse(data$X5.05.NYHA.dyspnoea.status..Pre.procedure..stable.only.=="Symptoms at rest or minimal activity", 1, 0)

 CCS.Class4.x <- ifelse(data$X5.04.CCS.angina.status..Pre.procedure..stable.only.=="Symptoms at rest or minimal activity", 1, 0)

 LV.fair.x <- ifelse(data$X6.08.LV.function=="Fair (LVEF = 30-49%)", 1, 0)
 LV.poor.x <- ifelse(data$X6.08.LV.function=="Poor (LVEF <30%)", 1, 0)

 recent.MI.x <- ifelse(data$X3.05.Previous.MI.and.interval.between.procedure.and.last.MI=="No previous MI" |data$X3.05.Previous.MI.and.interval.between.procedure.and.last.MI=="MI > 90 days", 0, 1)

 pul.hyp.yes.x <- ifelse(data$X6.012.PA.systolic.pressure..mmHg.>55, 1, 0)

 pul.hyp.no.x <- ifelse(data$X6.012.PA.systolic.pressure..mmHg.>31 & data$X6.012.PA.systolic.pressure..mmHg.<=55, 1, 0)

 urgent.x <- ifelse(data$X7.06.Procedure.urgency=="Urgent", 1, 0)
 emergency.x <- ifelse(data$X7.06.Procedure.urgency=="Emergency", 1, 0)
 salvage.x <- ifelse(data$X7.06.Procedure.urgency=="Salvage", 1, 0)

 weight.intervention.x <- rep(1, dim(data)[1]) #always 1.

 Thoracic.aortic.surgery.x <- rep(0, dim(data)[1]) #assume no for all.

 design.mat <- matrix(c(rep(1, dim(data)[1]), age.x, gender.x, renal.impair.fair.x, renal.impair.poor.x, renal.impair.dialysis.x, Extra.arterio.x, poor.mobility.x, pre.surgery.x, chronic.lung.x, Active.endocarditis.X, critical.preop.x, diabetes.insulin.x, NYHA2.x, NYHA3.x, NYHA4.x, CCS.Class4.x, LV.fair.x, LV.poor.x, recent.MI.x, pul.hyp.yes.x, pul.hyp.no.x, urgent.x, emergency.x, salvage.x, weight.intervention.x, Thoracic.aortic.surgery.x), ncol=27, byrow=FALSE) #.

 coeffs <- c(-5.324537, 0.0285181, 0.2196434, 0.303553, 0.8592256, 0.6421508, 0.5360268, 0.2407181, 1.118599, 0.1886564, 0.6194522, 1.086517, 0.3542749, 0.1070545, 0.2958358, 0.5597929, 0.2226147, 0.3150652, 0.8084096, 0.1528943, 0.3491475, 0.1788899, 0.3174673, 0.7039121, 1.362947, 0.0062118, 0.6527205)

 LP <- design.mat%*%coeffs
 ESII <- (exp(LP)/(1+exp(LP)))*100
 return(ESII)
}

## STS Score R Code

STS.Score <- function(data){
 atrial.fib.x <- ifelse(data$X3.11.1.Atrial.fibrillation.flutter=="Yes",
 1, 0)

 age.func1.x <- pmax(data$Age.at.Op-50, 0)
 age.func2.x <- pmax(data$Age.at.Op-75, 0)
 age.by.reop.x <- ifelse(data$X4.01.1.Previous.CABG=="Yes" |data$X4.01.2.Previous.Valve.Op=="Yes" |data$X4.01.3.Other.op.requiring.opening.of.pericardium=="Yes", age.func1.x, 0)
 age.by.urgency.x <- ifelse(data$X7.06.Procedure.urgency=="Emergency" | data$X7.06.Procedure.urgency=="Salvage",age.func1.x, 0)
 age.byMVR.x <- rep(0, dim(data)[1]) #assume no for all.
 age.byMVRepair.x <- rep(0, dim(data)[1]) #assume no for all.

 height.cm <- data$X5.01.Height * 100 #turn height into cm.
 BSA <- 0.007184*(height.cm^(0.725))*(data$X5.02.Weight^(0.425)) #DuBois Method
 BSA.func1.x <- (pmax(1.4,pmin(2.6, BSA))-1.8)
 BSA.func2.x <- ((pmax(1.4,pmin(2.6, BSA))-1.8)^2)

 CHF.and.NO.NYHA.x <- rep(0, dim(data)[1]) #can only use NYHA=IV as a surrogate.
 CHF.and.NYHA.x <- ifelse(data$X5.05.NYHA.dyspnoea.status..Pre.procedure..stable.only.=="Symptoms at rest or minimal activity",1, 0) #can only use NYHA=IV as a surrogate.

 CLD.x <- ifelse(data$X3.06.History.of.pulmonary.disease=="COAD/emphysema" |data$X3.06.History.of.pulmonary.disease=="Asthma" |data$X3.06.History.of.pulmonary.disease=="Other significant pulmonary disease",2, 0) ##assuming all those with COAD or asthma have moderate chronic lung disease
 CLD.by.MVR.x <- rep(0, dim(data)[1]) #assume no for all
 CLD.by.MVRepair.x <- rep(0, dim(data)[1]) #assume no for all

 Creatinine.mg.dl <- round(data$X3.03.Creatinine/88.4,2) #mg/dL.
 creat.func.x <- ifelse(data$X3.041.On.dialysis=="Yes",0, pmax(0.5, pmin(Creatinine.mg.dl, 5)))

 Diabetes.noninsulin.x <- ifelse(data$X3.01.Diabetes=="Diabetes (dietary control)" | data$X3.01.Diabetes=="Diabetes (oral medicine)", 1, 0)
 Diabetes.insulin.x <- ifelse(data$X3.01.Diabetes=="Diabetes (insulin)", 1, 0)

 Dialysis.x <- ifelse(data$X3.041.On.dialysis=="Yes", 1, 0)
 Dialysis.by.MVR.x <- rep(0, dim(data)[1]) #assume no for all.
 Dialysis.by.MVRepair.x <- rep(0, dim(data)[1]) #assume no for all.

 EF <- ifelse(data$X6.08.LV.function=="Good (LVEF >=50%)", 60,
 ifelse(data$X6.08.LV.function=="Fair (LVEF = 30-49%)", 45,
 ifelse(data$X6.08.LV.function=="Poor (LVEF <30%)", 30, 50)))
 Ejection.fraction.x <- (pmax(50-EF, 0))

 Active.Endocarditis.x <- rep(0, dim(data)[1]) #Assume no for all.

 Female.x <- ifelse(data$X1.07.Sex=="Female", 1, 0)
 Female.by.MVR.x <- rep(0, dim(data)[1]) #assume no for all.
 Female.by.MVRepair.x <- rep(0, dim(data)[1]) #assume no for all.
 Female.by.BSA1.x <- ifelse(Female.x==1, (pmax(1.4,pmin(2.6, BSA))-1.8), 0)
 Female.by.BSA2.x <- ifelse(Female.x==1, ((pmax(1.4,pmin(2.6, BSA))-1.8)^2), 0)

 Hypertension.x <- rep(0, dim(data)[1])

 IABP.inotropes.x <- ifelse(data$X5.031.Critical.pre.operative.status..v4.=="Yes", 1, 0)

 Immunosuppressive.treatment.x <- rep(0, dim(data)[1]) #missing so assume zero for all.

 LMD.x <- ifelse(data$X6.10.Left.main.stem.disease=="LMS >50% diameter stenosis", 1, 0)

 MI.within21days.x <- ifelse(data$X3.05.Previous.MI.and.interval.between.procedure.and.last.MI=="MI 6-24 hours" |data$X3.05.Previous.MI.and.interval.between.procedure.and.last.MI=="MI 1-30 days",1,0) #note <6 hours was grouped with 6-24 hours due to low patient numbers in former group.

 MVR.x <- rep(0, dim(data)[1]) #assume no for all.
 MVRepair.x <- rep(0, dim(data)[1]) #assume no for all.

 Peripheral.vascular.disease.x <- ifelse(data$X3.09.Extracardiac.arteriopathy=="Yes", 1, 0)
 re.op.x <- ifelse(data$X4.01.1.Previous.CABG=="Yes" |data$X4.01.2.Previous.Valve.Op=="Yes" |data$X4.01.3.Other.op.requiring.opening.of.pericardium=="Yes", 1, 0)

 shock.x <- ifelse(data$X5.031.Critical.pre.operative.status..v4.=="Yes", 1, 0)

 urgent.x <- ifelse(data$X7.06.Procedure.urgency=="Urgent", 1, 0)
 emergent.x <- ifelse(data$X7.06.Procedure.urgency=="Emergency", 1, 0)
 salvage.x <- ifelse(data$X7.06.Procedure.urgency=="Salvage", 1, 0)
 Status.by.MVR.x <- rep(0, dim(data)[1]) #assume no for all.
 Status.by.MVRepair.x <- rep(0, dim(data)[1]) #assume no for all.

 mitral.stenosis.x <- rep(0, dim(data)[1]) #assume none for all patients.

 CCS34 <- ifelse(data$X5.04.CCS.angina.status..Pre.procedure..stable.only.=="Marked limitation of ordinary physical activity" |data$X5.04.CCS.angina.status..Pre.procedure..stable.only.=="Symptoms at rest or minimal activity", 1, 0)
 Urgent <- ifelse(data$X7.06.Procedure.urgency=="Urgent" |data$X7.06.Procedure.urgency=="Emergency", 1, 0)
 unstable.angina.x <- ifelse(CCS34==1 & Urgent==1,
 ifelse(data$X3.05.Previous.MI.and.interval.between.procedure.and.last.MI=="MI 6-24 hours", 0, 1), 0)
 rm(CCS34); rm(Urgent)

 design.mat <- matrix(c(rep(1, dim(data)[1]), atrial.fib.x, age.func1.x, age.func2.x, age.by.reop.x, age.by.urgency.x, age.byMVR.x, age.byMVRepair.x, BSA.func1.x, BSA.func2.x, CHF.and.NO.NYHA.x, CHF.and.NYHA.x, CLD.x, CLD.by.MVR.x, CLD.by.MVRepair.x, creat.func.x, Diabetes.noninsulin.x, Diabetes.insulin.x, Dialysis.x, Dialysis.by.MVR.x, Dialysis.by.MVRepair.x, Ejection.fraction.x, Active.Endocarditis.x, Female.x, Female.by.MVR.x, Female.by.MVRepair.x, Female.by.BSA1.x, Female.by.BSA2.x, Hypertension.x, IABP.inotropes.x, Immunosuppressive.treatment.x, LMD.x, MI.within21days.x, MVR.x, MVRepair.x, Peripheral.vascular.disease.x, re.op.x, shock.x, urgent.x, emergent.x, salvage.x, Status.by.MVR.x, Status.by.MVRepair.x, mitral.stenosis.x, unstable.angina.x), ncol=45, byrow=FALSE)

 coeffs <- c(-5.78680, 0.18074, 0.03557, 0.02804, -0.01308, -0.02495, 0.01436, 0.02326, -1.40168, 2.16782, 0.2559, 0.60544, 0.23846, -0.15906, -0.03243, 0.43909, 0.23563, 0.48368, 1.48666, 0.4755, 0.78385, 0.00904, 0.66737, 0.20372, -0.10089, -0.23812, 0.96491, 0.18084, 0.11372, 0.38682, 0.35022, 0.17593, 0.13276, 0.10284, -0.6544, 0.21980, 0.74484, 0.47961, 0.25552, 1.32597, 2.07144, -0.31729, 0.84051, 0.21309, 0.18950)

 LP <- design.mat%*%coeffs
 STS <- (exp(LP)/(1+exp(LP)))*100
 return(STS)
}

## German AV R Code

German.AV.Score <- function(data){

 BMI <- ((data$X5.02.Weight)/(data$X5.01.Height^2))

 Age.66to70.x <- ifelse(data$Age.at.Op>=66 & data$Age.at.Op<=70, 1, 0)
 Age.71to75.x <- ifelse(data$Age.at.Op>=71 & data$Age.at.Op<=75, 1, 0)
 Age.76to80.x <- ifelse(data$Age.at.Op>=76 & data$Age.at.Op<=80, 1, 0)
 Age.81to85.x <- ifelse(data$Age.at.Op>=81 & data$Age.at.Op<=85, 1, 0)
 Age.greater85.x <- ifelse(data$Age.at.Op>85, 1, 0)

 Female.x <- ifelse(data$X1.07.Sex=="Female", 1, 0)

 BMI.less22.x <- ifelse(BMI<22, 1, 0)
 BMI.greater35.x <- ifelse(BMI>35, 1, 0)

 NYHA.Class4 <- ifelse(data$X5.05.NYHA.dyspnoea.status..Pre.procedure..stable.only.=="Symptoms at rest or minimal activity", 1, 0)

 MI.x <- ifelse(data$X3.05.Previous.MI.and.interval.between.procedure.and.last.MI=="No previous MI" |data$X3.05.Previous.MI.and.interval.between.procedure.and.last.MI=="MI 31-90 days" | data$X3.05.Previous.MI.and.interval.between.procedure.and.last.MI=="MI > 90 days", 0, 1)

 Critical.Preop.x <- ifelse(data$X5.031.Critical.pre.operative.status..v4.=="Yes", 1, 0)

 Hypertension.x <- ifelse(data$X6.01.PA.systolic...60mmHg=="Yes", 1, 0)

 No.sinus.rhytm.x <- ifelse(data$X3.11.2.Sinus.Rhythm=="No", 1, 0)

 LVEF.30to50.x <- ifelse(data$X6.08.LV.function=="Fair (LVEF = 30-49%)", 1, 0)
 LVEF.less30.x <- ifelse(data$X6.08.LV.function=="Poor (LVEF <30%)", 1, 0)

 Endocarditis.x <- rep(0, dim(data)[1])

 Previous.Heart.surg.x <- ifelse(data$X4.01.1.Previous.CABG=="Yes" |data$X4.01.2.Previous.Valve.Op=="Yes" |data$X4.01.3.Other.op.requiring.opening.of.pericardium=="Yes", 1, 0)

 Arterial.vessel.disease.x <- ifelse(data$X3.09.Extracardiac.arteriopathy=="Yes", 1, 0)

 COPD.x <- ifelse(data$X3.06.History.of.pulmonary.disease=="COAD/emphysema" |data$X3.06.History.of.pulmonary.disease=="Asthma" |data$X3.06.History.of.pulmonary.disease=="Other significant pulmonary disease", 1, 0)

 Dialysis.x <- ifelse(data$X3.03.Creatinine>200 | data$X3.041.On.dialysis=="Yes",1,0)

 Emergency.x <- ifelse(data$X7.06.Procedure.urgency=="Emergency" | data$X7.06.Procedure.urgency=="Salvage",1,0)

 design.mat <- matrix(c(rep(1, dim(data)[1]), Age.66to70.x, Age.71to75.x, Age.76to80.x, Age.81to85.x, Age.greater85.x, Female.x, BMI.less22.x, BMI.greater35.x, NYHA.Class4, MI.x, Critical.Preop.x, Hypertension.x, No.sinus.rhytm.x, LVEF.30to50.x, LVEF.less30.x, Endocarditis.x, Previous.Heart.surg.x, Arterial.vessel.disease.x, COPD.x, Dialysis.x, Emergency.x), ncol=22, byrow=FALSE)
 coeffs <- c(-5.504, 0.461, 0.909, 1.292, 1.782, 2.351, 0.357, 0.359, 0.393, 0.532, 0.825, 0.662, 0.398, 0.343, 0.283, 0.570, 0.545, 0.307, 0.359, 0.318, 1.164, 1.057)
 LP <- design.mat%*%coeffs
 German.AV.Score <- (exp(LP)/(1+exp(LP)))*100
 return(German.AV.Score)
}

## FRANCE-2 R Code

Iung.TAVI.Score <- function(data){
 BMI <- ((data$X5.02.Weight)/(data$X5.01.Height^2))

 Age.x <- ifelse(data$Age.at.Op>=90, 1, 0)

 BMI.18to30.x <- ifelse(BMI>=18.5 & BMI<=29.9, 1, 0)
 BMI.LESS18 <- ifelse(BMI<18.5, 1, 0)

 Respiratory.Insuffic.x <- ifelse(data$X3.06.History.of.pulmonary.disease=="COAD/emphysema" |data$X3.06.History.of.pulmonary.disease=="Asthma" |data$X3.06.History.of.pulmonary.disease=="Other significant pulmonary disease", 1, 0)

 Acute.Pulmonary.Oedema.x <- rep(0, dim(data)[1]) #assume no for all.

 NYHA.Class4 <- ifelse(data$X5.05.NYHA.dyspnoea.status..Pre.procedure..stable.only.=="Symptoms at rest or minimal activity", 1, 0)

 Dialysis.x <- ifelse(data$X3.041.On.dialysis=="Yes", 1,0)

 Hypertension.x <- ifelse(data$X6.01.PA.systolic...60mmHg=="Yes", 1, 0)

 Critical.preop.state.x <- ifelse(data$X5.031.Critical.pre.operative.status..v4.=="Yes", 1, 0)

 Transapical.access.x <- ifelse(data$X7.10.Delivery.approach=="Transapical", 1, 0)

 Other.access.x <- ifelse(data$X7.10.Delivery.approach=="Direct aortic" | data$X7.10.Delivery.approach=="Other" | data$X7.10.Delivery.approach=="Subclavian", 1, 0)

 design.mat <- matrix(c(rep(1, dim(data)[1]), Age.x, BMI.18to30.x, BMI.LESS18, Respiratory.Insuffic.x, Acute.Pulmonary.Oedema.x, NYHA.Class4 , Dialysis.x, Hypertension.x, Critical.preop.state.x, Transapical.access.x, Other.access.x), ncol=12, byrow=FALSE)
 coeffs <- c(-3.32, 0.42, 0.41, 0.82, 0.50, 0.47, 0.58, 1.06, 0.37, 0.87, 0.70, 0.78)
 LP <- design.mat%*%coeffs
 Lung.TAVI.Score <- (exp(LP)/(1+exp(LP)))*100
 return(Lung.TAVI.Score)
}

## OBSERVANT R Code

OBSERVANT.TAVI.score <- function(data){
 GlomerularFiltrationRate <- ifelse(data$X1.07.Sex=="Female",ifelse(data$X1.08.Ethnic.origin=="Other", (175*((data$X3.03.Creatinine/88.4)^-1.154)*(data$Age.at.Op^(-0.203))*(0.742)*(1.212)), (175*((data$X3.03.Creatinine/88.4)^-1.154)*(data$Age.at.Op^(-0.203))*(0.742))),

ifelse(data$X1.08.Ethnic.origin=="Other", (175*((data$X3.03.Creatinine/88.4)^-1.154)*(data$Age.at.Op^(-0.203))*(1.212)), (175*((data$X3.03.Creatinine/88.4)^-1.154)*(data$Age.at.Op^(-0.203)))))

GFR.less45.X <- ifelse(GlomerularFiltrationRate <45, 6, 0)

 Critical.State.x <- ifelse(data$X5.031.Critical.pre.operative.status..v4.=="Yes", 5, 0)

 Hypertension.x <- ifelse(data$X6.01.PA.systolic...60mmHg=="Yes", 4, 0)

 Diabetes.x <- ifelse(data$X3.01.Diabetes=="Not Diabetic", 0, 4)

 NYHA.X <- ifelse(data$X5.05.NYHA.dyspnoea.status..Pre.procedure..stable.only.=="Symptoms at rest or minimal activity", 4, 0)

 Prior.BAV.x <- ifelse(data$X4.021.Balloon.aortic.valvuloplasty.prior.to.date.of.TAVI=="Yes" |data$X7.074.Aortic.balloon.valvuloplasty.before.valve.deployment=="Completed", 3, 0)

 LVEF.less40.x <- ifelse(data$X6.08.LV.function=="Good (LVEF >=50%)", 0, 3)

 LP <- GFR.less45.X + Critical.State.x + Hypertension.x + Diabetes.x + NYHA.X + Prior.BAV.x + LVEF.less40.x
 OBSERVANT.Score <- ((exp(-4+(0.15*LP)))/(1+exp(-4+(0.15*LP))))*100
 return(OBSERVANT.Score)
}

## ACC TAVI R Code

ACC.TAVI.Score <- function(data){

Age.x <- floor(data$Age.at.Op/5)

GlomerularFiltrationRate <- ifelse(data$X1.07.Sex=="Female",ifelse(data$X1.08.Ethnic.origin=="Other", (175*((data$X3.03.Creatinine/88.4)^-1.154)*(data$Age.at.Op^(-0.203))*(0.742)*(1.212)), (175*((data$X3.03.Creatinine/88.4)^-1.154)*(data$Age.at.Op^(-0.203))*(0.742))),

ifelse(data$X1.08.Ethnic.origin=="Other", (175*((data$X3.03.Creatinine/88.4)^-1.154)*(data$Age.at.Op^(-0.203))*(1.212)), (175*((data$X3.03.Creatinine/88.4)^-1.154)*(data$Age.at.Op^(-0.203))))) #Modification of Diet in Renal Disease formula

GlomerularFiltrationRate <- floor(GlomerularFiltrationRate/5)

Dialysis.x <- ifelse(data$X3.041.On.dialysis=="Yes", 1,0)

NYHA.Class4 <- ifelse(data$X5.05.NYHA.dyspnoea.status..Pre.procedure..stable.only.=="Symptoms at rest or minimal activity", 1, 0)

Respiratory.Insuffic.x <- ifelse(data$X3.06.History.of.pulmonary.disease=="COAD/emphysema" |data$X3.06.History.of.pulmonary.disease=="Asthma" | data$X3.06.History.of.pulmonary.disease=="Other significant pulmonary disease", 1, 0)

NonTransfemoral.access.x <- ifelse(data$X7.10.Delivery.approach=="Femoral - percutaneous" |data$X7.10.Delivery.approach=="Femoral - surgical", 0, 1)

PreMI <- ifelse(data$X3.05.Previous.MI.and.interval.between.procedure.and.last.MI=="MI 6-24 hours", 1, 0)

AcuityCategory2 <- ifelse(data$X7.06.Procedure.urgency=="Urgent" & data$X5.031.Critical.pre.operative.status..v4.=="No" & PreMI==0, 1, 0)

AcuityCategory3 <- ifelse((data$X7.06.Procedure.urgency=="Urgent" | data$X7.06.Procedure.urgency=="Elective") & data$X5.031.Critical.pre.operative.status..v4.=="Yes" & PreMI==0, 1, 0)

AcuityCategory4 <- ifelse(data$X7.06.Procedure.urgency=="Emergency" | data$X7.06.Procedure.urgency=="Salvage" | PreMI==1, 1, 0)

design.mat <- matrix(c(rep(1, dim(data)[1]), Age.x, GlomerularFiltrationRate, Dialysis.x, NYHA.Class4, Respiratory.Insuffic.x , NonTransfemoral.access.x, AcuityCategory2, AcuityCategory3, AcuityCategory4), ncol=10, byrow=FALSE)

coeffs <- c(-4.72976, 0.12185, -0.06933, 1.17932, 0.22304, 0.51084, 0.67347, 0.45070, 0.99269, 1.20737)

LP <- design.mat%*%coeffs

ACC.TAVI.Score <- (exp(LP)/(1+exp(LP)))*100

return(ACC.TAVI.Score)

}
